# Pre-stack seismic inversion for reservoir characterization in Pleistocene to Pliocene channels, Baltim gas field, Nile Delta, Egypt

**DOI:** 10.1038/s41598-024-75015-x

**Published:** 2025-01-07

**Authors:** Ali S. El-Sayed, Walid M. Mabrouk, Ahmed M. Metwally

**Affiliations:** Geophysics Department, FacultyofScience, Cairo University, Giza, 12613 Egypt

**Keywords:** Pre-stack seismic inversion, EEI, Gas bearing sand, Baltim field, Offshore Nile Delta, Environmental sciences, Physics

## Abstract

The Nile Delta, North Africa’s leading gas-producing region, was the focus of this study aimed at delineating gas-bearing sandstone reservoirs from the Pleistocene to Pliocene formations using a combination of pre-stack inversion and rock physics analysis. This research employed seismic inversion techniques, including full-angle stack seismic volumes, well logs, and 3-D with rock physics modeling to refine volumes of P-wave velocity (Vp), S-wave velocity (Vs), and density. Traditional seismic attributes, such as far amplitude, proved insufficient for confirming gas presence, highlighting partial angle stacks, integrated the need for advanced methods. Extended Elastic Impedance (EEI) analysis was used to predict fluids and identify lithology in clastic reservoir environments. The EEI approach facilitated the determination of optimal projection angles for key petrophysical properties such as porosity, shale volume, and water saturation. This method was applied to the middle Pliocene (Kafr El Sheikh Formation) and the Pleistocene (El Wastani Formation), revealing promising drilling sites. In the Kafr El Sheikh Formation, porosity ranged from 16 to 29%, shale volume from 21 to 40%, and hydrocarbon saturation from 25 to 90%. The study concludes that integrating pre-stack seismic inversion with EEI significantly enhances the likelihood of identifying gas-bearing sands while reducing exploration risks. The improved POS for the Pleistocene anomaly gas bearing sand (from 49 to 69%) and the middle Pliocene anomaly (from 46 to 66%) underscores the effectiveness of this approach in the Baltim Field, Offshore Nile Delta, and supports further drilling and development wells.

## Introduction

The Baltim field, a major offshore Mediterranean gas deposit, was developed by IEOC and commenced production in late 1999. Covering an area of 576 km^2^, the field extends from a sea depth of 45 m in the south to 300 m in the north, situated approximately 7 km from the Mediterranean coastline^1–3^. Exploration began in the Mediterranean offshore in 1993–1994, with IEOC’s successful appraisal drilling at the Baltim East-1 well leading to further drilling^4,5^. Subsequently, IEOC, in collaboration with Amoco, drilled seven additional wells, discovering three distinct fields: Baltim North, East, and South^6,7^.

The first onshore well, Mit Ghamr-1, was drilled by IEOC in 1966, and the initial gas discovery was made in the Abu Madi-1 well in 1967^8^. The Nile Delta Basin is estimated to hold around 223 trillion cubic feet of gas across 126 reserves, with many of these fields containing mature source rocks similar to the Neogene deposits^9,10^. A significant portion of this gas is biogenic.

The offshore Nile Delta has rapidly become a significant gas production area, aided by high-quality 3-D seismic surveys and data from numerous deep-water exploration wells^11,12^. Notable fields in this region include Baltim Eastern and Northern, known for substantial gas condensate accumulations. The first gas reserves were indicated by the successful drilling of Baltim East-01 in 1993, with further confirmation provided by Baltim North-01 in 1995. To date, 12 wells have been drilled in the Baltim East area and seven in the Baltim North region^8,13^.

The Baltim field comprises two main geological formations: the Pleistocene El Wastani and the Pliocene Kafr El Sheikh. Modeling suggests that the Lower and Middle Pliocene periods are particularly conducive to biogenic gas formation, although the Miocene sequences are believed to have produced more biogenic gas compared to the Plio-Late Pliocene phases^14,15^.

Seismic inversion techniques, including post- and pre-stack inversion, are utilized to derive elastic rock properties from seismic data, translating seismic reflection amplitudes into impedance profiles^16–20^. This approach enhances seismic resolution and allows for more precise geological and reservoir characterization. However, the primary challenge lies in confirming the presence of gas in the Pleistocene and Pliocene anomalies, which complicates identification and increases the need for accurate predictions.

To address this, a combination of post- and pre-stack seismic inversion techniques, alongside Extended Elastic Impedance (EEI) analysis, is employed. EEI inversion provides critical elastic parameters such as water saturation, shale volume, and effective porosity. This approach not only confirms gas presence in the Kanaria anomaly but also significantly increases the probability of success (POS) and reduces drilling risks. By integrating these parameters, the study facilitates more accurate gas zone identification and enhances opportunities for successful exploration and development.

## Geologic setting of Nile delta

**Fig. 1:**
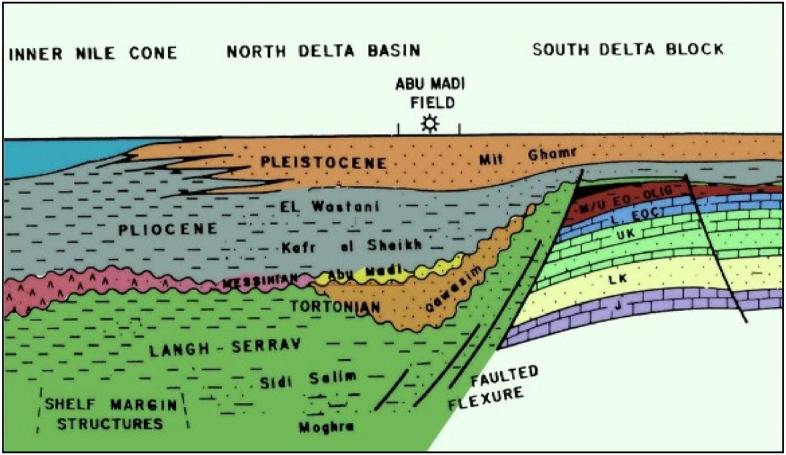
Scheme of the stratigraphic and facies relations between South Delta block and North Nile Delta basin^46,69^.

A prominent flexure zone is the hinge zone, often known as the hinge axis, which extends through the northern and southern Delta Basins, the two separate sections that make up the Nile Delta region^21,22^. These narrow zones are located approximately at 31 degrees’ latitude and exhibit an east–west orientation. It is important to emphasize that this boundary moreover operates as a facies limit for structural separation (Fig. 1).

**Fig. 2:**
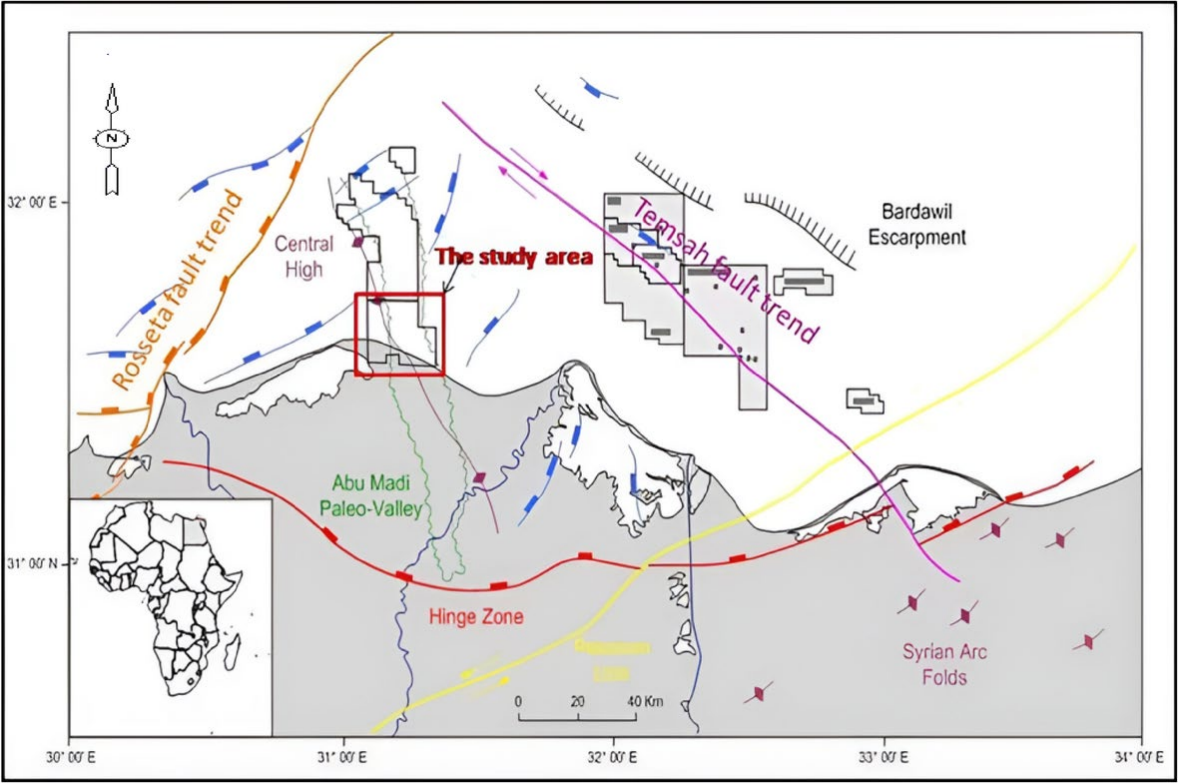
Nile Delta Regional Structural Framework^62^.

In their study, the tectonic activity has influenced the Nile Delta basin into three primary phases: The Late Cretaceous Period-Early Tertiary, Late Paleozoic—Early Mesozoic, and Late Eocene-Recent. As a consequence of these three tectonic phases, the Nile Delta is governed by six major first-order structural trends^21^, as depicted in (Fig. 2).

These trends include the following:

- The Pelusium Megashear structural trend, which extends in a northeast direction.
- The Neogene Hinge Line, which exhibits an east–west orientation.
- The Rosetta Fault Trend, which trends in a northeast direction.
- The Temsah Structural Trend, which trends northwest.
- The Red Sea—Gulf of Suez Fault Trend, which trends northwest.
- The Baltim Fault Trend, which trends north–south.

Several published research studies have focused on the tectonic setting, stratigraphy, and sedimentological characteristics of the Mediterranean Sea and Nile Delta regions. The sedimentary portion in the Nile Delta is situated within the Precambrian African craton’s crystalline basement. The deposition of sediments within the northern Delta Embayment is attributed to the accumulation of sand particles derived from terrestrial sources that can be traced back to the Precambrian, Paleozoic, and Mesozoic eras^23,24^.

Within the Delta Province, it is discovered three sedimentary phases that span from the middle Miocene to the Holocene^22,25^ (Fig. 1). The initial cycle, known as the Miocene cycle, encompasses the formations of Sidi Salim, Qawasim, Abu Madi, and Rosetta. The Mit Ghamr Formations, El Wastani Formations, and Kafr El Sheikh Formations are part of the second cycle (the Pliocene cycle). The Bilqas Formation is a representation of the third cycle, or the Recent cycle^26–29^. Formations of Qawasim as well as Abu Madi are considered to be components of a single regressive period during the late Miocene era, following the transgressive Sidi Salim Formation^30^. Furthermore, the Early Pliocene sea level rise cycle represented by the Kafr El Sheikh Formation was considered a transgression.

The Nile Delta’s geological framework is shaped by its prominent structural features and complex stratigraphy. Structurally, the Delta is divided into northern and southern basins, intersected by major fault trends such as the Pelusium Megashear, Rosetta Fault, and Baltim Fault, among others^16,31^. These structural elements have evolved through distinct tectonic phases, including the Late Cretaceous-Early Tertiary and Late Eocene-Recent periods, resulting in significant regional geological formations^21^.

**Fig. 3:**
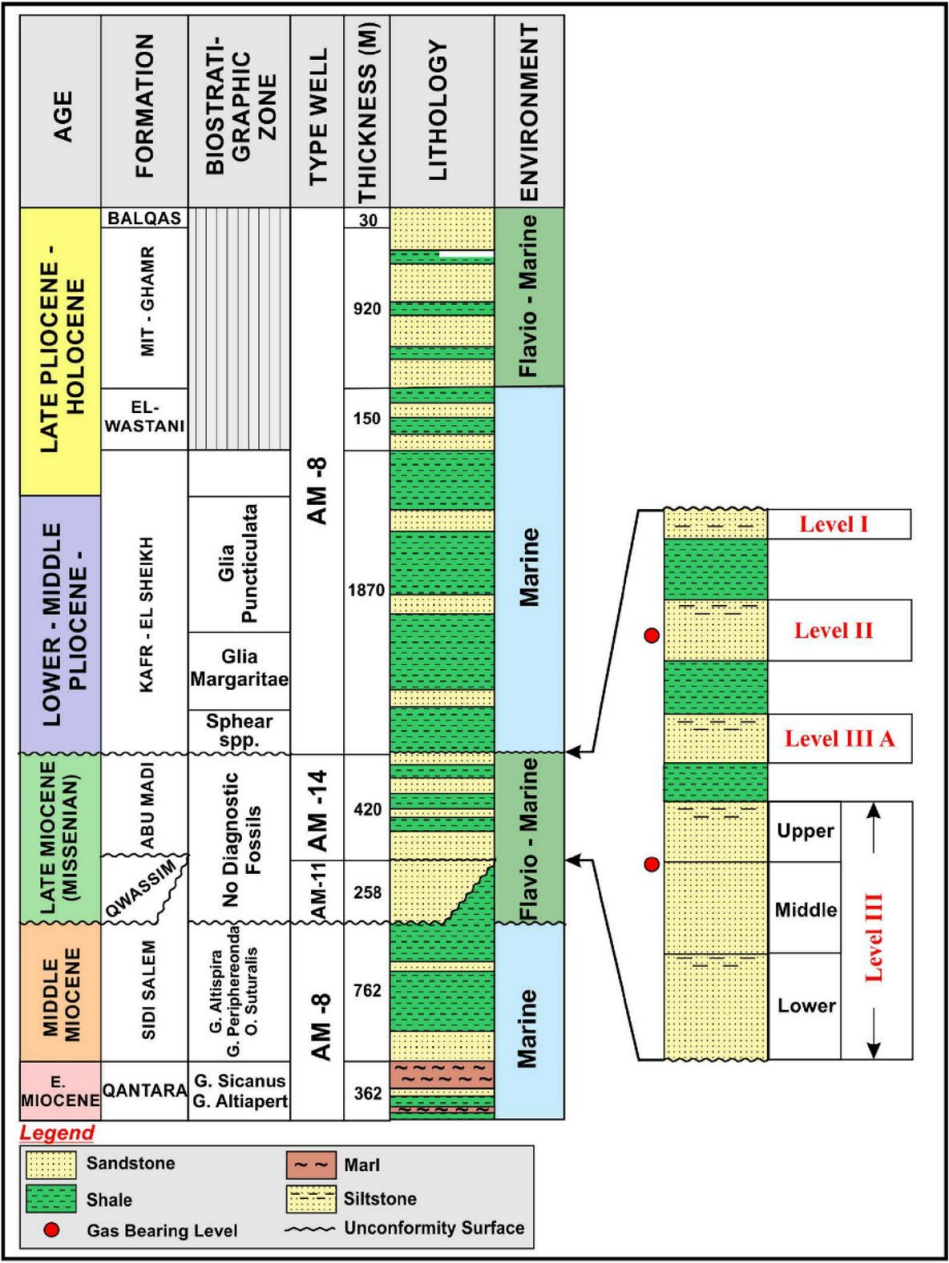
Stratigraphic column of Baltim Field in the Nile Delta^48^.

The stratigraphy of the Nile Delta, extending from the Precambrian to the Holocene, is characterized by a series of sedimentary cycles. The Miocene cycle includes formations like Sidi Salim and Qawasim, while the Pliocene cycle features the El Wastani and Kafr El Sheikh Formations. The recent cycle is represented by the Bilqas Formation (Fig. 3)^26–29^.

**Fig. 4:**
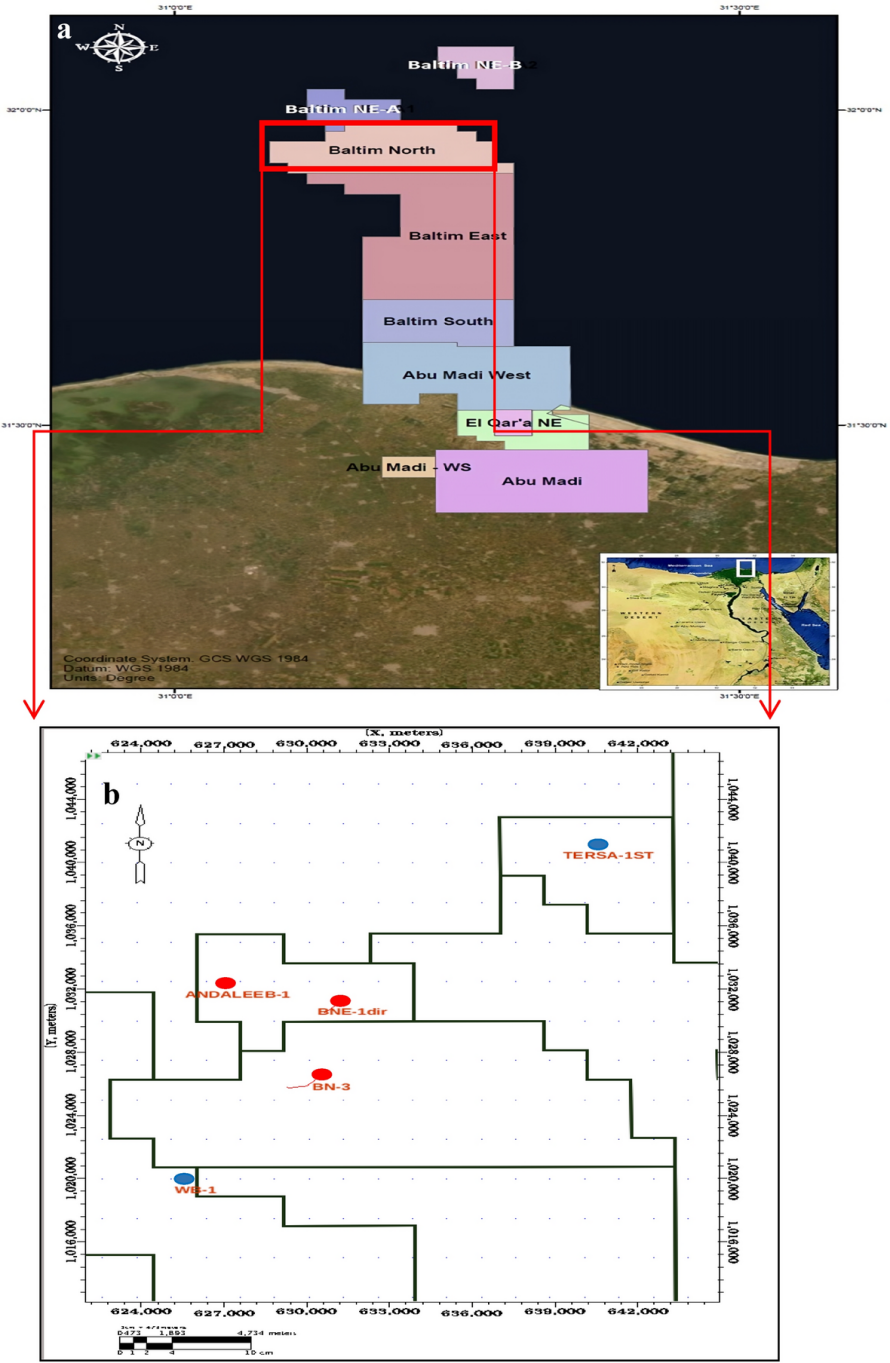
(**a**) Location map of a central Nile Delta, Egypt. (**b**) Base map showing the well locations.

In the Baltim area, the focus is on the Kanaria prospect, which encompasses two significant anomalies: the Pleistocene anomaly (El Wastani Formation) and the Pliocene anomaly (Kafr El Sheikh Formation). The El Wastani Formation, associated with the Pleistocene anomaly, exhibits distinctive seismic responses due to gas effects, which are crucial for identifying the top gas sands and interpreting subsurface structures. Similarly, the Kafr El Sheikh Formation, linked to the Pliocene anomaly, contributes to understanding the deeper stratigraphy and hydrocarbon potential in the region. By incorporating a detailed examination of these formations and their geological context, this study provides a more comprehensive view of the factors influencing seismic data and petrophysical analyses in the Nile Delta. The analysis of these formations is integral to understanding the regional geology’s impact on seismic data interpretation and petrophysical analysis, offering insights into their role in hydrocarbon accumulation and migration, and thereby enhancing the overall comprehension of the Nile Delta’s geological context (Fig. 4).

## Methodology

Seismic inversion is a technique used to minimize the impact of the wavelet on seismic data through deconvolution, followed by adjustments to derive impedance. This study specifically focuses on pre-stack inversion and Extended Elastic Impedance (EEI), both of which are deterministic methods aimed at producing a single, highly accurate model of the subsurface. These methods provide valuable insights into lithological and fluid properties by analyzing seismic data.

The seismic inversion approach in this study is characterized as deterministic, reflecting the use of well-defined models to derive subsurface properties directly from seismic data. This classification highlights the precision of the methodology in deriving subsurface models. The combination of seismic characteristics, Wireline log evaluation, pre-stack inversion, and EEI contributes to seismic reservoir characterization, a well-established technique for understanding and quantifying hydrocarbon reserves.

Impedance plays a crucial role in seismic analysis for several reasons. It is the most effective metric for detecting lateral lithological changes and is essential for detailed hydrocarbon analysis and exploration, particularly in offshore fields. Additionally, impedance is used to calculate the reflection coefficient, which is derived from normalized variations in acoustic impedance along reflecting boundaries. Importantly, impedance serves as a non-geologic property that can be directly interpreted in geological terms.

**Fig. 5:**
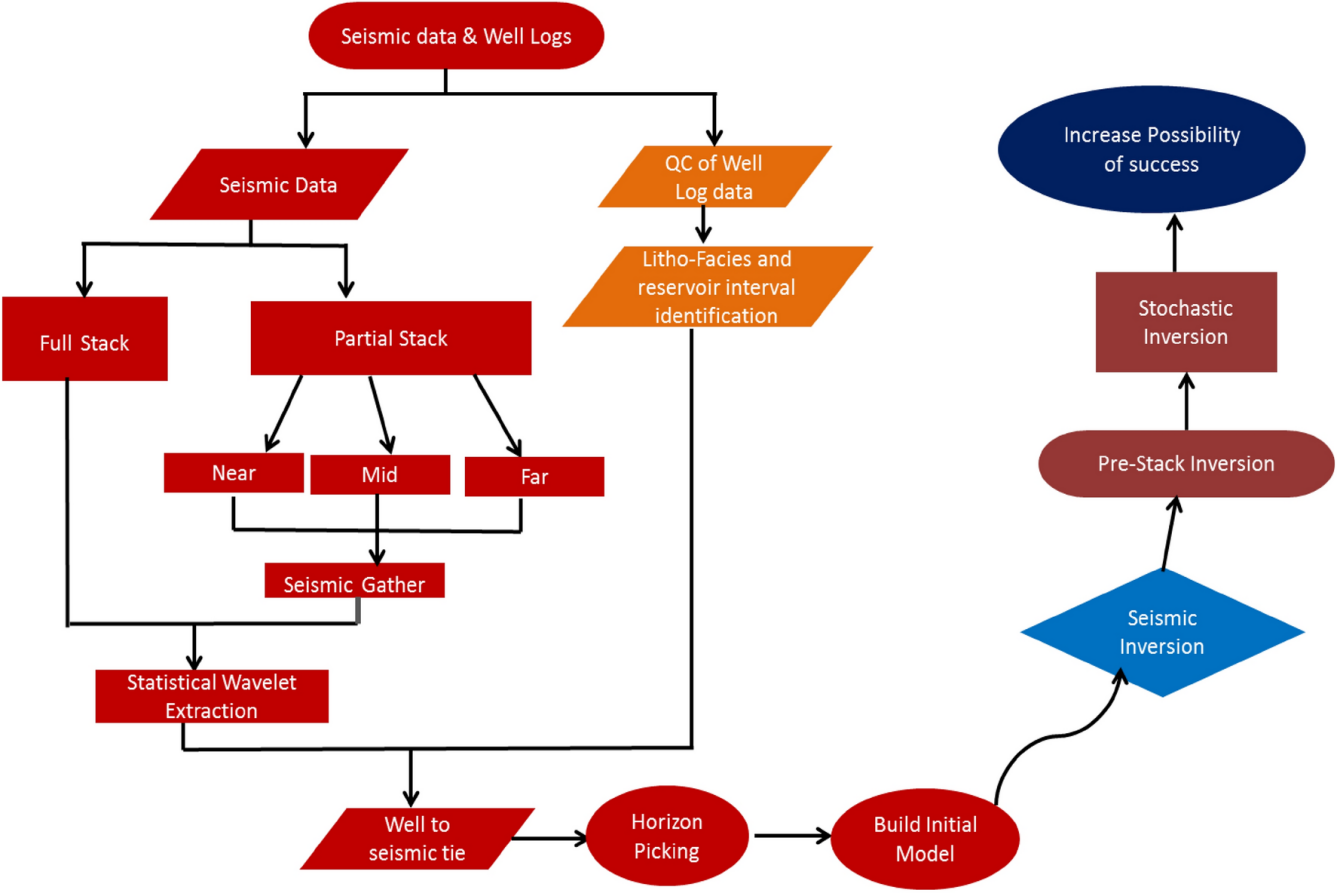
Research Workflow, From Seismic data till risk assessment.

The study employs various seismic inversion methods. Post-stack inversion transforms a full-stack seismic volume into acoustic impedance by integrating seismic and well data with structural and stratigraphic information. This method involves analyzing different frequency components derived from seismic velocity and well data. Pre-stack inversion, on the other hand, utilizes angle-dependent data for amplitude versus offset (AVO) analysis to derive parameters related to P-impedance, S-impedance, and rock density. Extended Elastic Impedance (EEI) combines P-wave velocity (VP), S-wave velocity (VS), and density in an angle-dependent manner. The workflow from seismic data and well logs till seismic inversion is summarized (Fig. 5)

For this study, shear wave data from wells such as WB-1 and BNE-1 were incorporated into the EEI process. Partial stack data, including near, mid, and far stacks, supported the pre-stack inversion and EEI analysis. Cross plots showing the relationships between acoustic impedance and shear impedance, color-coded by gas saturation, shale volume, water saturation, and porosity, further support this approach. Additional details are discussed in the QC for Well Logs section.

Alignment of seismic and well data was achieved by applying filters to the wavelets, adjusting both frequency and time components. This filtering process ensured that the seismic and well data were correctly aligned for accurate analysis. The filtered data was then used in an extensive investigation of seismic inversion results from both post- and pre-stack data, along with EEI. This approach improved the assessment of reservoir characteristics and increased the likelihood of identifying gas-bearing sands.

Seismic inversion suppresses the wavelet’s impact on seismic data using deconvolution, then adjusts what emerges to obtain impedance.

The inversion process in this study is focused on pre-stack inversion and Extended Elastic Impedance (EEI), which are deterministic methods. These methods aim to derive a single, most accurate model of the subsurface, providing precise insights into lithological and fluid properties through seismic data analysis.

Based on the concluded output, the seismic inversion method in this study is classified as deterministic. This classification reflects the approach of deriving subsurface properties directly from seismic data using well-defined models.

With the combination of seismic characteristics, Wireline log evaluation, as well as pre-stack inversion and EEI, seismic reservoir characterization is a well-known technique for gaining insight and quantifying hydrocarbon reserves^32^. Impedance plays a critical role in seismic analysis for several reasons^33,34^:

- Identification of lithological changes: Impedance is the best metric for detecting lateral lithological changes.
- Hydrocarbon analysis and exploration: It is useful for in-depth analysis of hydrocarbon fields and offshore exploration.
- Reflection coefficient calculation: The reflection coefficient is computed using normalized variations in acoustic impedance along reflecting borders.
- Geological interpretation: Impedance is a non-geologic property that can be directly interpreted geologically.

Seismic inversion methods include:

- Post-stack inversion: Transforms a full stack seismic volume into acoustic impedance by integrating seismic and well data, along with structural and stratigraphic information^31,35^. It involves various frequency components derived from seismic velocity and well data^14^.
- Pre-stack inversion: Utilizes angle-dependent data for AVO analysis to derive parameters related to P-impedance, S-impedance, and rock density^36–38^.

Extended Elastic Impedance (EEI) integrates P-wave velocity (VP), S-wave velocity (VS), and density in an angle-dependent manner. Shear wave data from wells such as WB-1 and BNE-1 were utilized in the EEI process. Partial stack data, including near, mid, and far stacks, were gathered to support the pre-stack inversion and EEI analysis. Cross plots showing relationships between acoustic impedance and shear impedance, colored by gas saturation, shale volume, water saturation, and porosity, support this approach. These details are further discussed in the QC for Well Logs section.

Seismic and well data alignment was achieved by applying filters to the wavelets, adjusting both frequency and time components. This filtering process ensured that the seismic and well data were correctly aligned for accurate analysis. The filtered data was then used in an extensive investigation to analyze seismic inversion results from both post- and pre-stack data, along with Extended Elastic Impedance (EEI). This approach enhanced the assessment of reservoir characteristics and improved the chances of identifying gas-bearing sands (Fig. 5).

## Available data

The study systematically organized seismic data into a structured grid format, focusing on a 3D structural grid within the Baltim Concessions (Fig. 4). This organization included data from five drilled wells aligned with the seismic grid and pre-stack seismic data at three reflection angles: near, mid, and far. Seismic volumes were arranged into four-angle stacks—full, near (0°–15°), mid (15°–30°), and far (30°–45°)—to enable a comprehensive interpretation of subsurface features. The primary objective was to apply pre-stack inversion and Extended Elastic Impedance (EEI) to enhance the understanding of anomalies in the Kanaria area, particularly within the middle and late Pliocene formations. This approach aimed to predict elastic parameters such as porosity, gas saturation, and shale volume, thereby increasing the confidence in identifying gas-bearing sand reservoirs.

Additionally, well logs utilized in this study included resistivity, gamma ray, and density logs. These inputs were examined to determine key petrophysical parameters, including shale volume (VSh), total porosity (ΦT), effective porosity (ΦE), and water saturation (Sw), which are critical for assessing hydrocarbon potential.

## Results

### Seismic interpretation and petrophysical analysis

#### Seismic interpretation

**Fig. 6:**
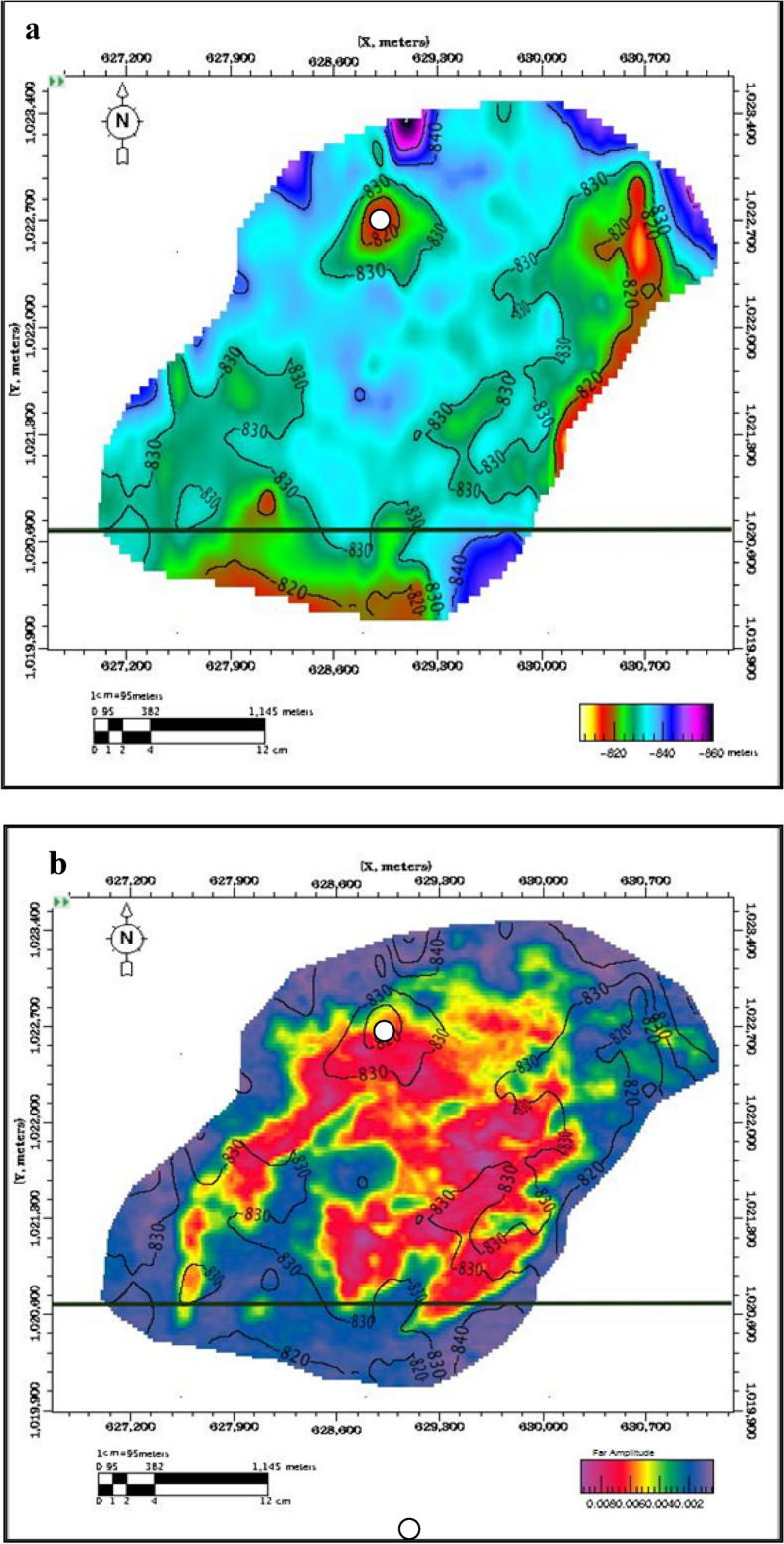
(**a**) Pleistocene Anomaly, Structure Depth Map, PSTM. (**b**) Pleistocene Anomaly, (− 25, 50) ms, far Amp./Depth Map.

**Fig. 7:**
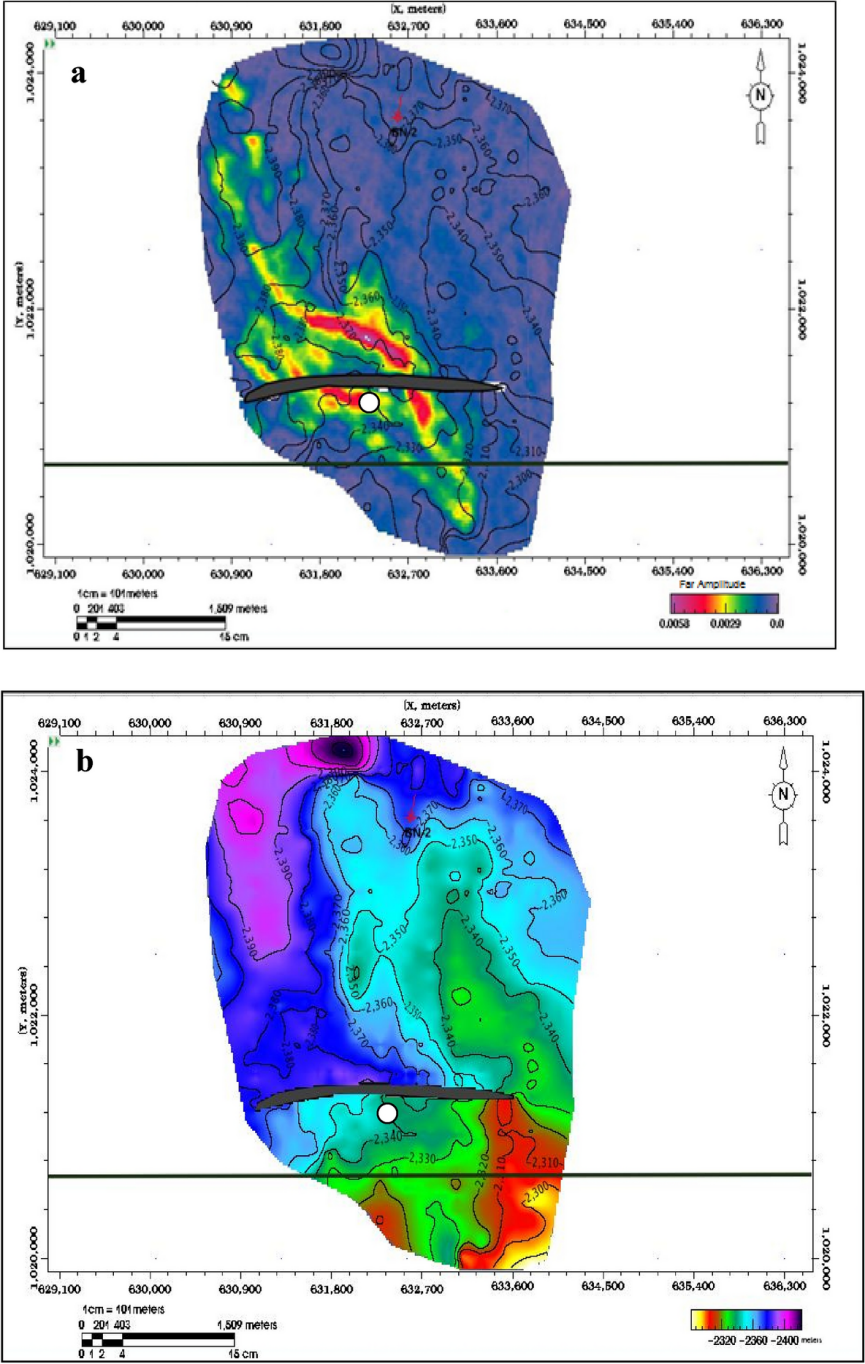
(**a**) Middle Pliocene Anomaly, Structure Depth Map, PSTM. (**b**) Pleistocene Anomaly, (− 10, 40) ms, far Amp./Depth Map.

Seismic data was utilized to interpret the regional structural geometry, identify various faults, and select horizons^39–41^. Synthetic seismograms were generated for the Pleistocene anomaly (El Wastani Fm.) at well Wb-1 and for the Upper Pliocene anomaly (Kafr El Sheikh Fm.) at wells BNE-1 dir and ANDALEEB-1. A time grid for the El Wastani Fm. was constructed with a velocity of 1800 m/s, resulting in a depth contour map ranging from 810 to 850 m (Fig. 6). For the Kafr El Sheikh Fm., a single velocity function of 2070 m/s indicated a depth range from 2300 to 2380 m (Fig. 7).

#### Petrophysical analysis

**Fig. 8:**
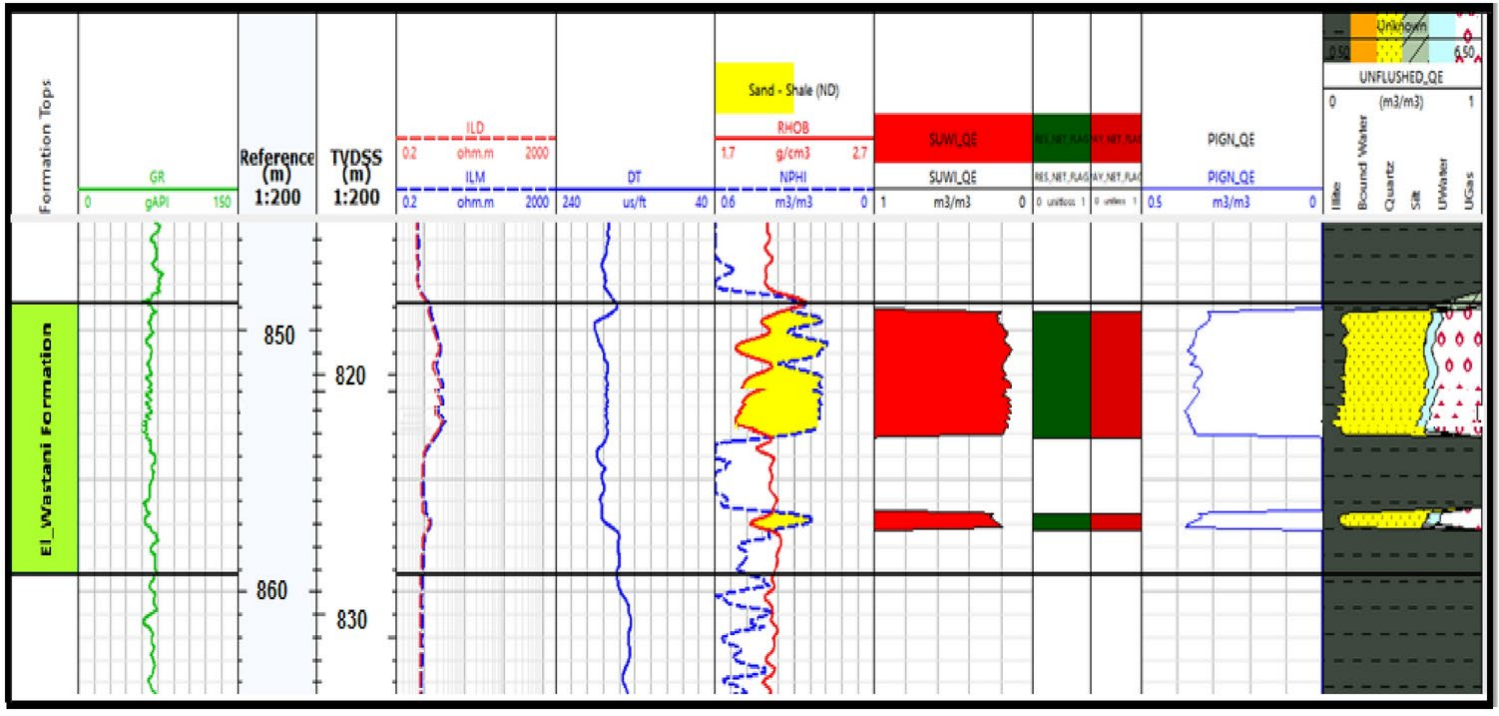
Petrophysical interpretation of WB-1 dir well (EL Wastani Fm.)

**Fig. 9:**
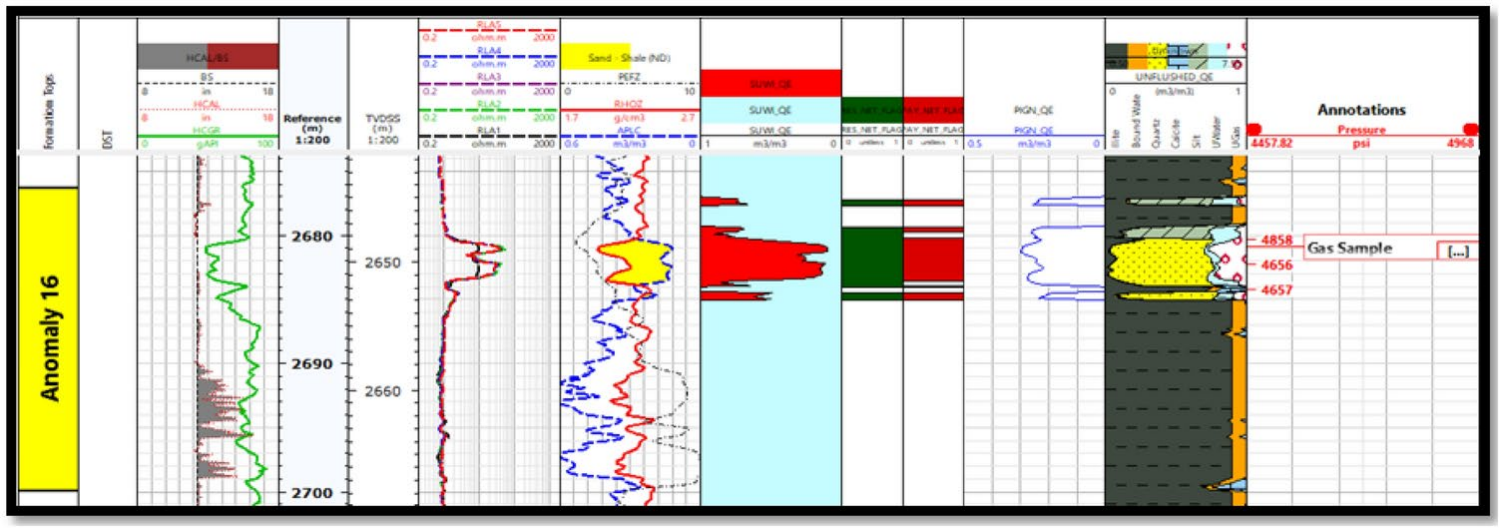
Petrophysical interpretation of ANDALEEB-1 dir well (Kafr El sheikh Fm.)

Petrophysical evaluation was conducted on the Pleistocene anomaly (El Wastani Formation) and the Pliocene anomaly (Kafr El Sheikh Formation), both primarily sandstone reservoirs (Figs. 8 and 9). The analysis covered the El Wastani Fm. from 816 to 826 meters and the Kafr El Sheikh Fm. from 2644 to 2668 meters to quantify the reservoirs. Key parameters assessed include:

- Porosity (Φ): Total porosity (ΦTPHIT) for the El Wastani Fm. ranges from 16 to 36%, with effective porosity (Φ_E_) between 12 and 29%. For the Kafr El Sheikh Fm., Φ_T_ ranges from 21 to 32% at BNE-1 dir and 21 to 29% at ANDALEEB-1, with Φ_EPHIE_ spanning from 12 to 29% and 15 to 29%, respectively.
- Shale Volume (V_Sh_) and Water Saturation (S_w_): These were calculated using numerical techniques and volumetric methods, considering factors such as clay typing, formation water salinity, and mineral modeling.

Petrophysical analysis was conducted at the El Wastani Fm. (Pleistocene Anomaly) and the Kafr El Sheikh Fm. (Middle Pliocene Anomaly), which spans from 816 to 826 m for the El Wastani Formation and from 2644 to 2668 m at the Kafr El Sheikh Formation, in order to quantify the reservoir. The distinct crossover produced by resistivity logs indicates an abundance of hydrocarbons in the reservoir. The intersection of porosity values obtained from neutron curves with bulk density log curves allows for the most accurate identification of the porous zones.

The Dual Water shaly sand assessment approach was employed to address the reservoir characteristics. This method involves creating a model with an additional conductivity parameter, explaining the absence of electrical charges in the clay’s diffuse surrounding layer. The Dual Water method evaluates two crucial factors: the cementation factor (m) and the saturation component (n), which are influenced by the rock’s porosity or tortuosity. The salinity of the formation water was determined using a Pickett plot created from SP log data, with the dual water saturation prediction model applying m = 1.69 and n = 2.0 for sandstone intervals, despite core data^5,33,42–46^. The petrophysical parameters of the two wells are summarized at (Table 1).

**Table 1:** Summarized petrophysical parameters for WB-1 and ANDALEEB-1 Wells.

Parameters	West Baltim-1 (WB-1)		ANDALEEB-1	
Intervals	MD. (m)	TVDss. (m)	MD. (m)	TVDss. (m)
Gross thickness	959–969	926–236	2670–2700	2644–2668
Net reservoir	10	10	24	24
Net pay	4	4	6	6
Por. range (res.)	4	4	6	5
Sw. range (res.)	(30–36) Av. 32%		(21–29) Av. 25%	
Por. range (pay)	(18–25) Av. 18%		(10–75) Av. 41%	
Sw. range (pay)	(30–36) Av. 32%		(16–27) Av. 24%	

**Fig. 10:**
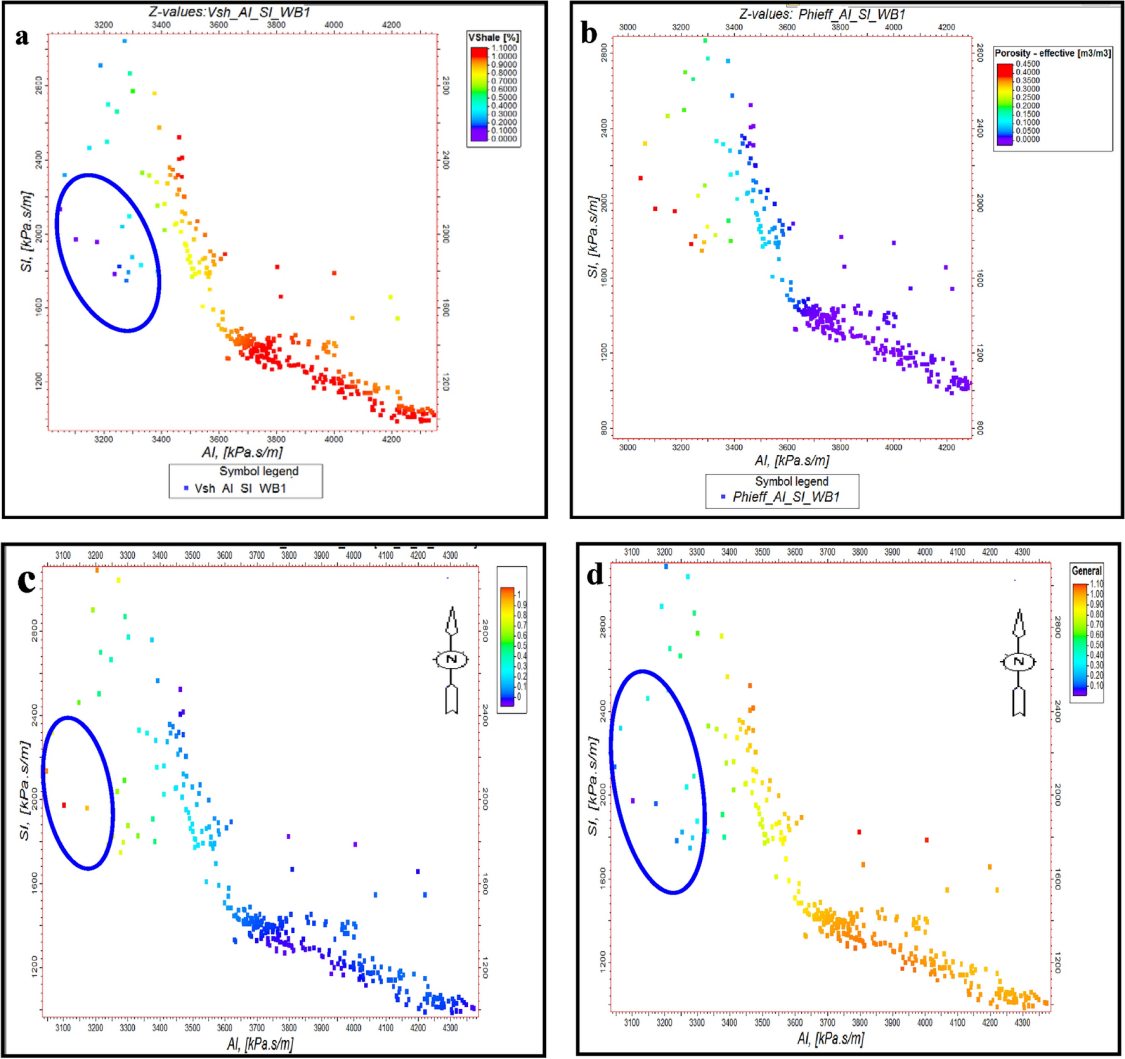
(**a**) AI Versus SI, with Vsh, as plotting curve from Neural Net at WB-1 well (El Wastani Fm.). (**b**) AI Versus SI, with Phieff, as plotting curve from Neural Net at WB-1 well (El Wastani Fm.). (**c**) AI Versus SI, with Sg, as plotting curve from Neural Net at WB-1 well (El Wastani Fm.). (**d**) AI Versus SI, with Sw, as plotting curve from Neural Net at WB-1 well (El Wastani Fm.)

**Fig. 11:**
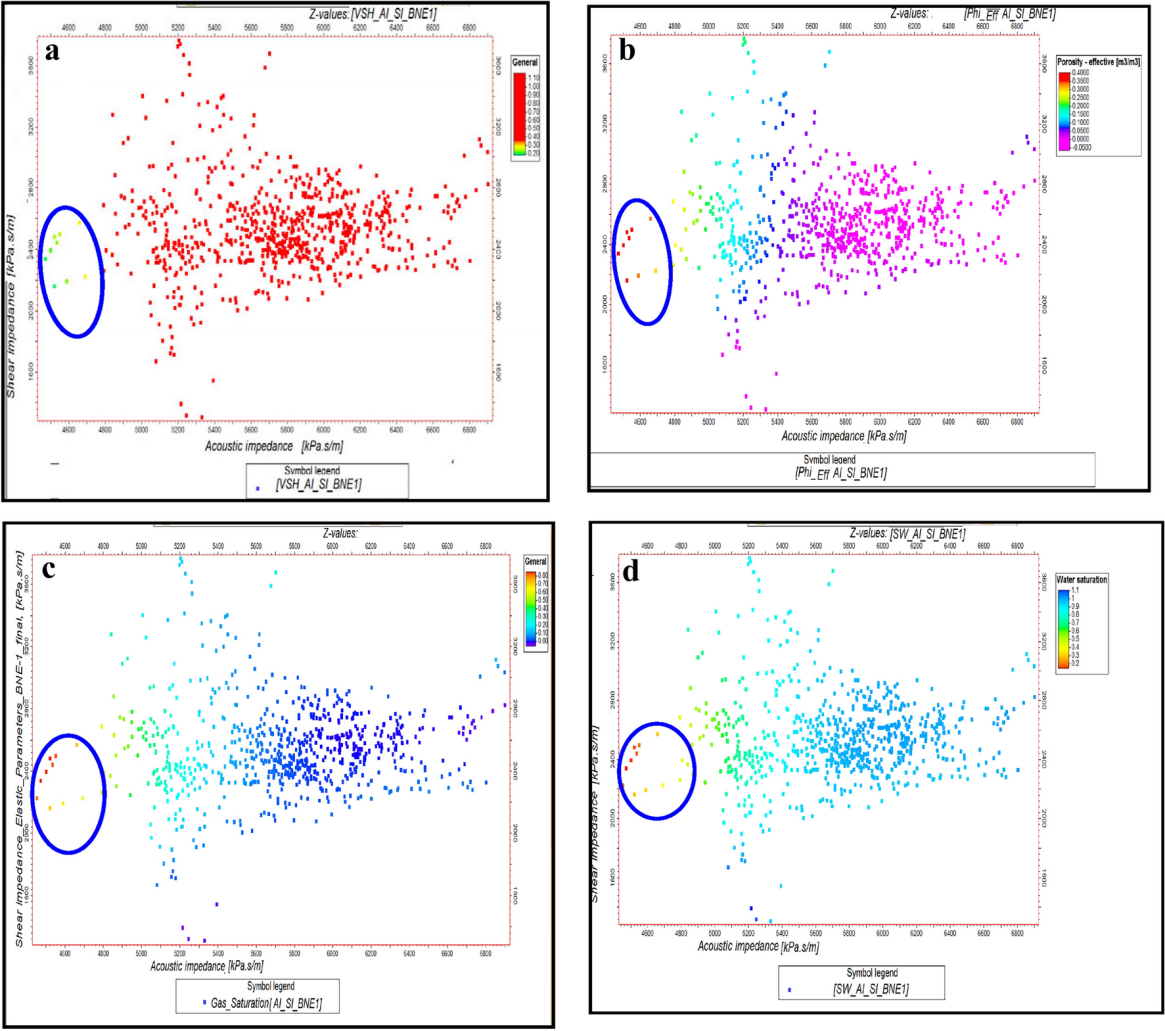
(**a**) AI Versus SI, with Vsh, as plotting curve from Neural Net at BNE-1 dir well (Kafr El Shiek Fm.). (**b**) AI Versus SI, with PHIeff, as plotting curve from Neural Net at BNE-1 dir well (Kafr El Shiek Fm.). (**c**) AI Versus SI, with Sg, as plotting curve from Neural Net at BNE-1 dir well (Kafr El Shiek Fm.). (**d**) AI Versus SI, with Sw, as plotting curve from Neural Net at WB-1 well (El Wastani Fm.)

Outliers in Fig. 10 correspond to the presence of gas zones, characterized by low acoustic impedance and low shear impedance values at WB-1 key well for Pleistocene anomaly. These outliers are cross-plotted with shale volume, effective porosity and gas saturation data to validate their geological significance. The identification of gas zones and other geological features was refined using elastic parameter estimates and rock physics correlations. At the WB-1 well, acoustic and shear impedance, along with Poisson’s ratio, were used to distinguish between shale, wet sand, and gas sand. This was visualized by plotting acoustic impedance (AI) against shear impedance (SI), with additional charts showing relationships between volume of shale, porosity, water saturation, and gas saturation at BNE-1 dir key well for Pliocene anomaly (Figs. 10 and 11). Isolated points with low AI and SI, highlighted in blue circles, indicate gas zones.

**Fig. 12:**
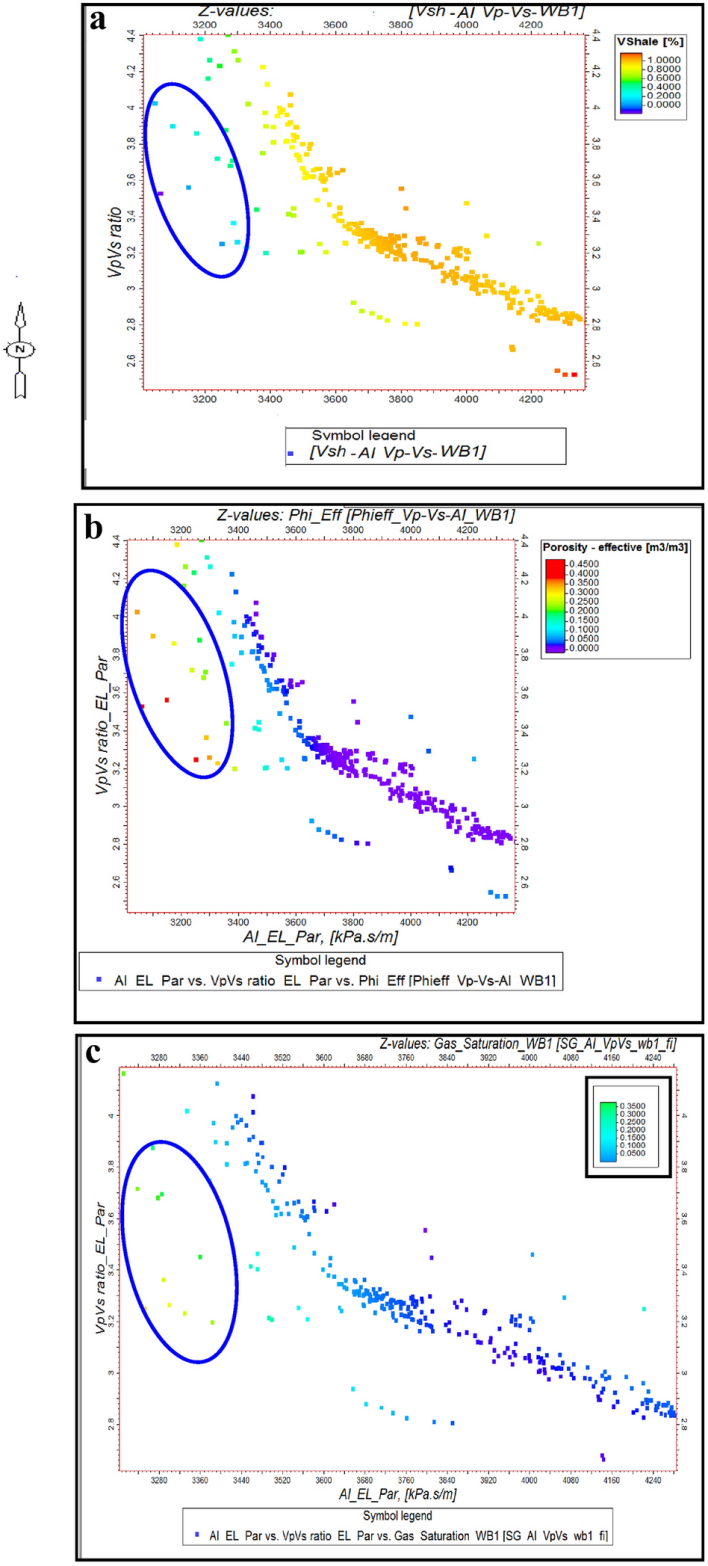
(**a**) AI Versus Vp/Vs ratio, with Vsh, as plotting curve from Neural Net at WB-1 well (El Wastani Fm.). (**b**) AI Versus Vp/Vs ratio, with PHIeff, as plotting curve from Neural Net at WB-1 well (El Wastani Fm.). (**c**) AI Versus Vp/Vs ratio, with Sg, as plotting curve from Neural Net at WB-1well (El Wastani Fm.).

**Fig. 13:**
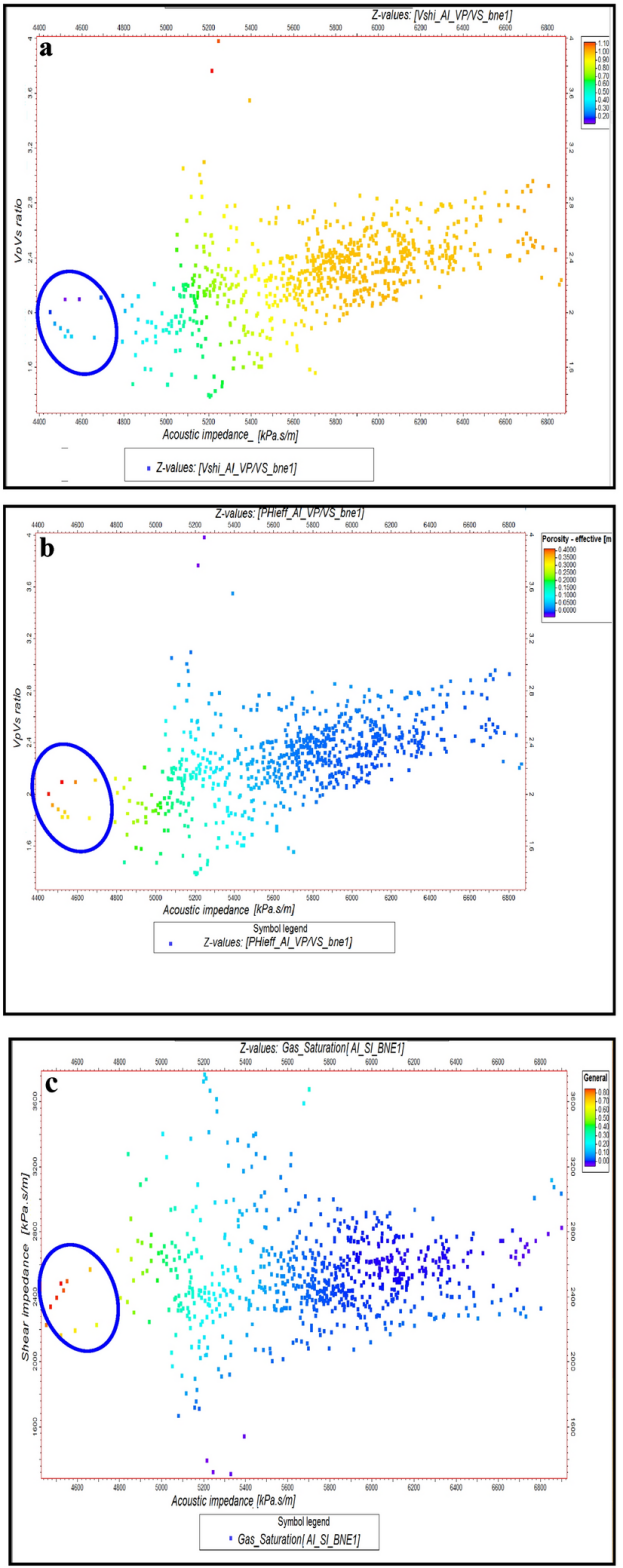
(**a**) AI Versus Vp/Vs ratio, with Vsh, as plotting curve from Neural Net at BNE-1 dir well (Kafr El Shiek Fm.). (**b**) AI Versus Vp/Vs ratio, with PHIeff, as plotting curve from Neural Net at BNE-1 dir well (Kafr El Shiek Fm.). (**c**) AI Versus Vp/Vs ratio, with Sg, as plotting curve from Neural Net at BNE-1 dir well (Kafr El Shiek Fm.).

Similarly, at well BNE-1, these parameters were used to analyze the second Pliocene anomaly, showing isolated points in low AI and high Vp/Vs regions as indicative of gas zones. Further, rock physics relationships at both wells allowed for differentiation between shale and sand types (wet or gas) by plotting shale volume, porosity, and gas saturation against AI and Vp/Vs ratio (Figs. 12 and 13). These methods effectively highlight outliers in the data, reflecting the presence of gas zones and enhancing the geological interpretation. This analysis helps in understanding the distribution and impact of gas in the reservoir.

### Seismic inversion and synthetic seismograms

#### Synthetic seismograms

Synthetic seismograms were generated from well logs and combined with actual field seismic data for calibration. Synthetic data are created by modeling seismic responses based on well logs, simulating how the seismic waves would interact with the subsurface if we had perfect information. Notably, the synthetic seismograms utilized zero-phase and European polarity, which are crucial for accurate amplitude interpretation. Zero-phase wavelets minimize phase distortions, ensuring that the seismic data more accurately reflect the true subsurface properties. European polarity affects the amplitude responses of the seismic data, aiding in the clear distinction between different geological features. These factors are essential for improving the identification of gas-bearing sands by providing a more precise interpretation of the subsurface conditions.

Composite data, on the other hand, combine this synthetic data with actual field seismic data collected from the exploration area. By integrating both types of data, the analysis benefits from a more comprehensive dataset, allowing for better calibration and interpretation.

Seismic inversion techniques utilize seismic data and well logs to model rock properties and fluids. Even without well data, lithology and fluid parameters can be inferred from seismic inversion^32,47–54^. A critical aspect of seismic interpretation is correlating well data (depth units) with seismic data (time units)^34,47,50,55^. This correlation enables linking the reservoir layers in the El-Wastani and Kafr El Sheikh Formations observed in wells with reflections in the seismic section. Acoustic impedance is derived from density and P-sonic measurements.

**Fig. 14:**
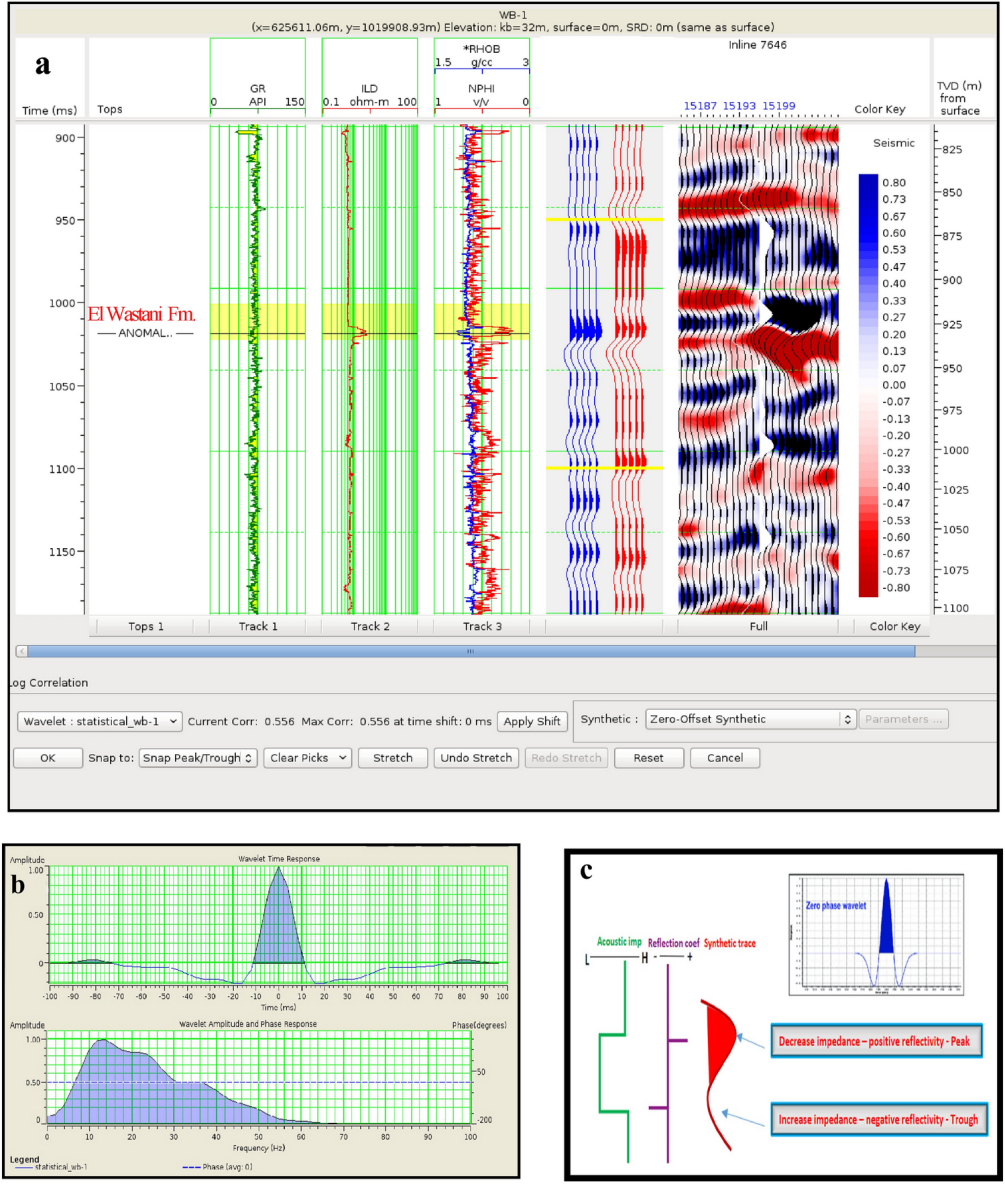
(**a**) Well to seismic tie for WB-1 well. (**b**) Statistical wavelet of WB-1 well. (**c**) Phase and polarity of WB-1 well.

**Fig. 15:**
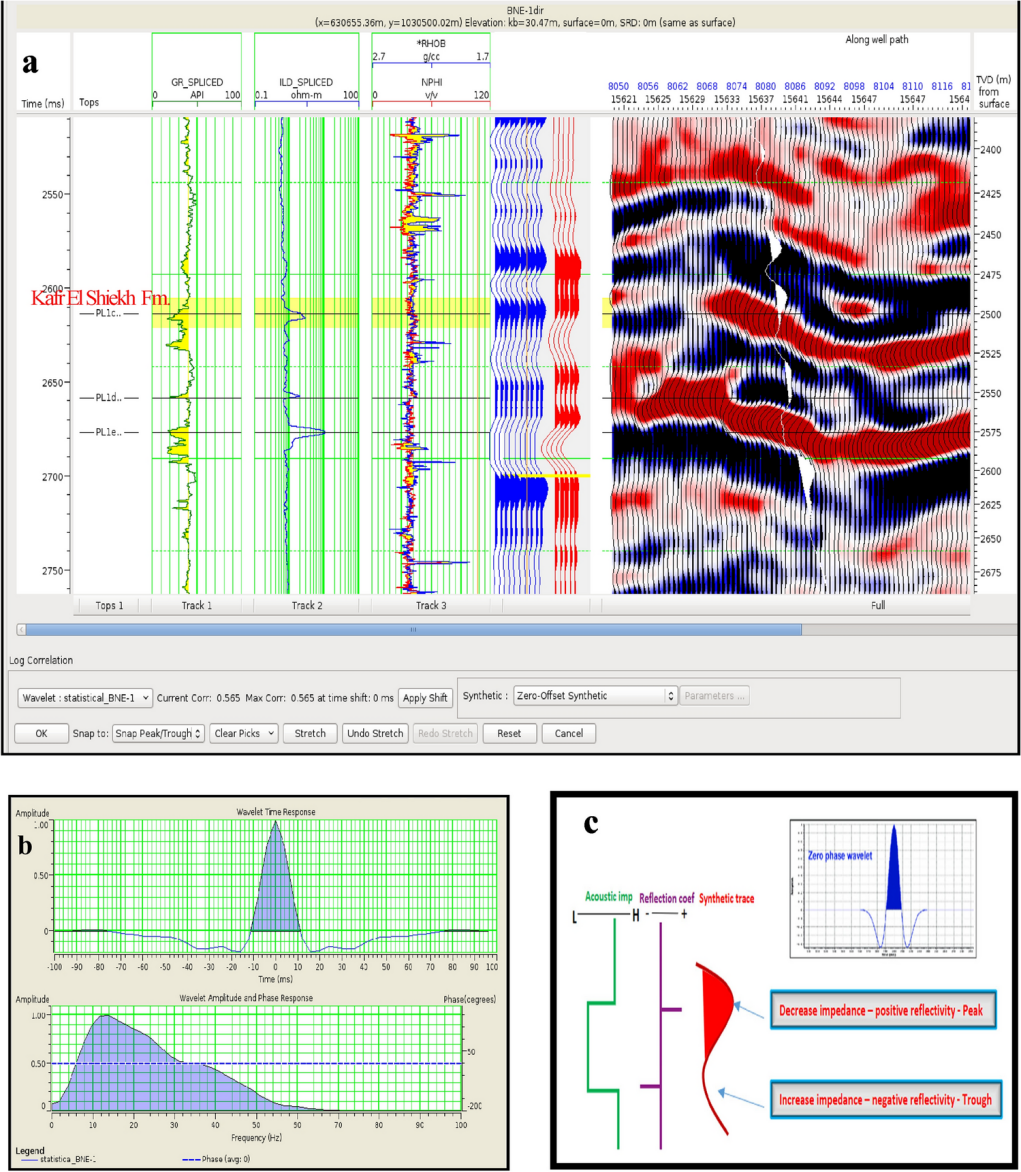
(**a**) Well to seismic tie for BNE-1 dir well. (**b**) Statistical wavelet of BNE-1 dir well. (**c**) Phase and polarity of BNE-1 dir well.

The analysis focused on two anomalies: the Pleistocene anomaly in well WB-1, with a correlation coefficient of 0.55% (Fig. 14), and the Pliocene anomaly in well BNE-1, with a correlation coefficient of 0.56% (Fig. 15). The results demonstrate the effectiveness of integrating synthetic and field data in correlating seismic data with well data from the five wells examined.

#### Seismic inversion

Seismic inversion was applied using Hampson–Russel and Petrel software, with additional risk assessment performed using Pres software. The workflow involved:

1. Quality Control (QC) of log and seismic data for both anomalies.
2. Seismic-to-well tie and wavelet extraction.
3. Initial model building for pre-stack inversion.
4. Execution of pre-stack inversion.
5. Extended Elastic Impedance (EEI) calculation.
6. Rock physics characterization.
7. Risk assessment.

#### Inversion analysis

Seismic inversion is a crucial technique for converting seismic reflection data into quantitative rock-property information. It involves the use of well logs and seismic data to create models of subsurface properties such as acoustic impedance, shear impedance, and density. Inversion methods allow for the interpretation of lithology and fluid content even in the absence of extensive well data, providing a valuable tool for understanding subsurface geology^19,55–57^.

In this study, several inversion techniques were applied to refine the initial impedance models, including both post-stack and pre-stack inversion approaches to ensure a comprehensive analysis. The inversion process began with the quality control (QC) of well logs and seismic data, followed by seismic-to-well tie and wavelet extraction. Initial models were constructed for pre-stack inversion, and the analysis was extended through the calculation of Extended Elastic Impedance (EEI), rock physics characterization, and risk assessment.

#### Comparison with well logs

**Fig. 16:**
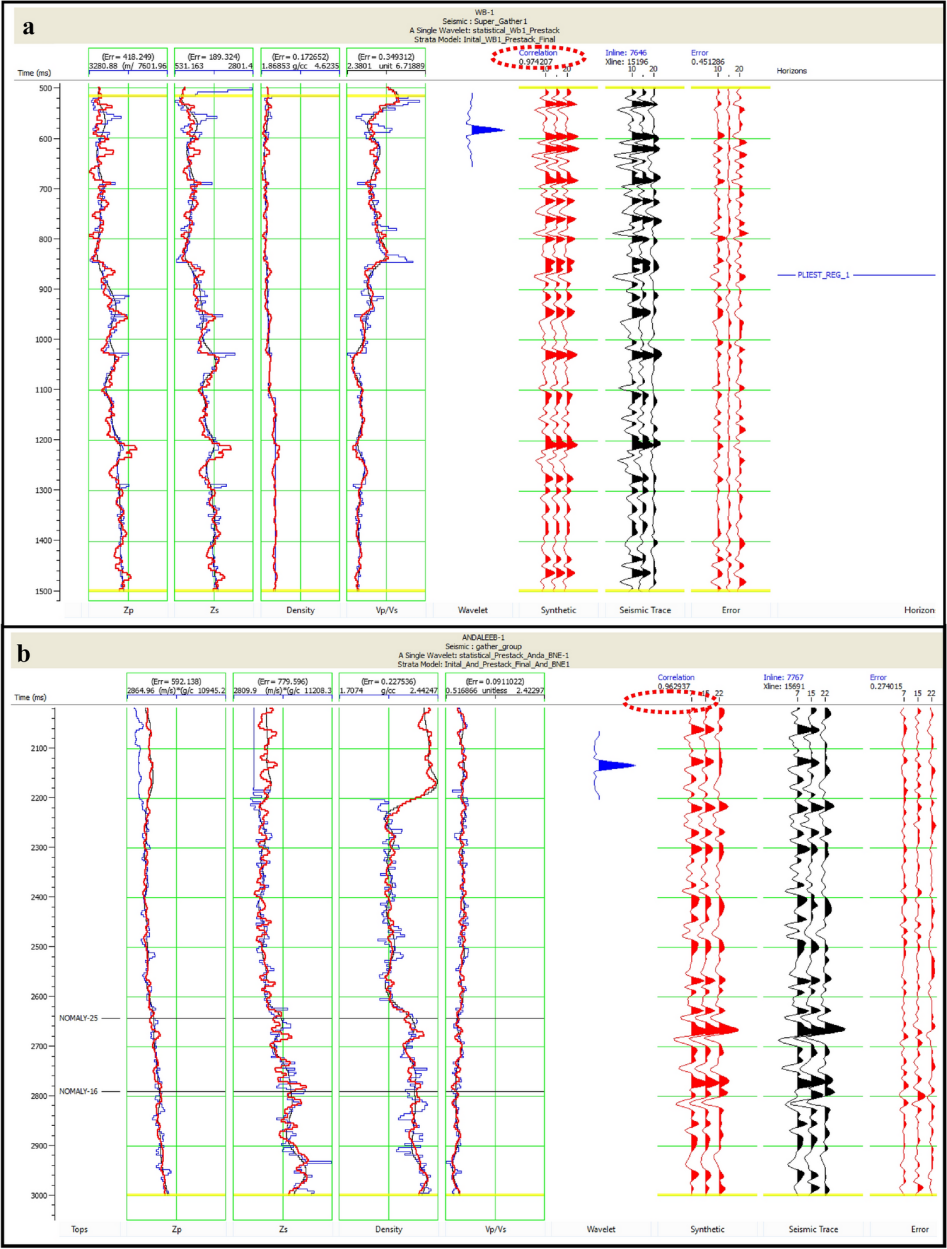
(**a**) Pre seismic inversion analysis, for WB-1 well, Pleistocene anomaly, with correlation approximately 97%. (**b**) Pre seismic inversion analysis, for Andaleeb-1 well, Pleistocene anomaly, with correlation approximately 96%.

Inversion analysis was performed for both anomalies identified in the study. For the Pleistocene anomaly, well WB-1 was used as the key well, resulting in an inversion analysis with a correlation coefficient of approximately 97% (Fig. 16a). This high correlation underscores the reliability of the inversion results in accurately reflecting the subsurface properties observed in WB-1. For the Pliocene anomaly, well ANDALEEB-1 served as the key well, achieving an impressive correlation of around 96% (Fig. 16b). These high correlations underscore the reliability of the inversion results in accurately reflecting the subsurface properties observed in the wells.

To validate the accuracy of the inversion results, synthetic seismograms (red-colored curves) were cross-correlated with actual seismic stack data (black-colored curves). The red curve represents the inverted acoustic impedance, while the blue curve corresponds to the original log data. The fit between the synthetic and seismic data is depicted by the black curve, illustrating the effectiveness of the inversion in matching the seismic reflections with the well log data.

Regarding the feasibility of the inversion, the frequency spectrum content in the study area ranged from 10 to 30 Hz. This frequency range was chosen to optimize the resolution and accuracy of the inversion results while minimizing phase distortions through the use of zero-phase wavelets. The inversion employed European polarities, which are crucial for aligning the phase of the seismic data with the inversion models. Evaluating the impact of frequency content and phase changes, in the context of European polarities, is essential for assessing the reliability of the inversion and ensuring that the results are robust against potential distortions.

In addition to the inversion techniques and analyses described, it is essential to align the seismic inversion results with geological concepts to enhance the credibility and relevance of the findings. This alignment ensures that the inversion outcomes are not only statistically accurate but also geologically meaningful. By integrating geological interpretations with the inversion results, including parameters such as water saturation, effective porosity, gas saturation, and shale volume, we validate that the modeled subsurface properties correspond with known geological structures and rock types. This approach confirms that the inversion results reflect the true geological setting of the study area, reinforcing the reliability of the interpretations derived from the seismic data. This comprehensive view helps in understanding how the inversion models translate into practical geological insights and ensures that the results support the broader geological framework of the study area.

### Pre stack inversion (simultaneous seismic inversion)

Pre-stack inversion (simultaneous seismic inversion) is performed using seismic data of the pre-stack type^58,59^. This inversion process simultaneously converts pre-stack gathers into P-wave velocity (Vp), S-wave velocity (Vs), and density. When the angle range exceeds 35 degrees, the derived bulk density can be considered reliable. This approach leverages the entirety of the pre-stack seismic data, yielding more precise and dependable results.

Advantages of pre-stack inversion:

- The output seismic data exhibits a higher degree of detail due to the reduction in thin bed and tuning effects, resulting from the removal of the seismic wavelet.

Disadvantages of pre-stack inversion:

- Seismic resolution limitations: The method is constrained by the seismic resolution, particularly in the interpretation of thin beds under the resolution limit, which may lead to less accurate results.
- Lateral shifts: The accuracy of the inversion may be affected by lateral variations in seismic data, which can introduce uncertainties in the interpretation.

Additionally, uncertainties associated with seismic inversion, such as the influence of noise, the quality of the input data, and assumptions made during the inversion process, are inherent challenges. These factors can impact the reliability of the results, particularly when dealing with complex geological settings.

**Fig. 17:**
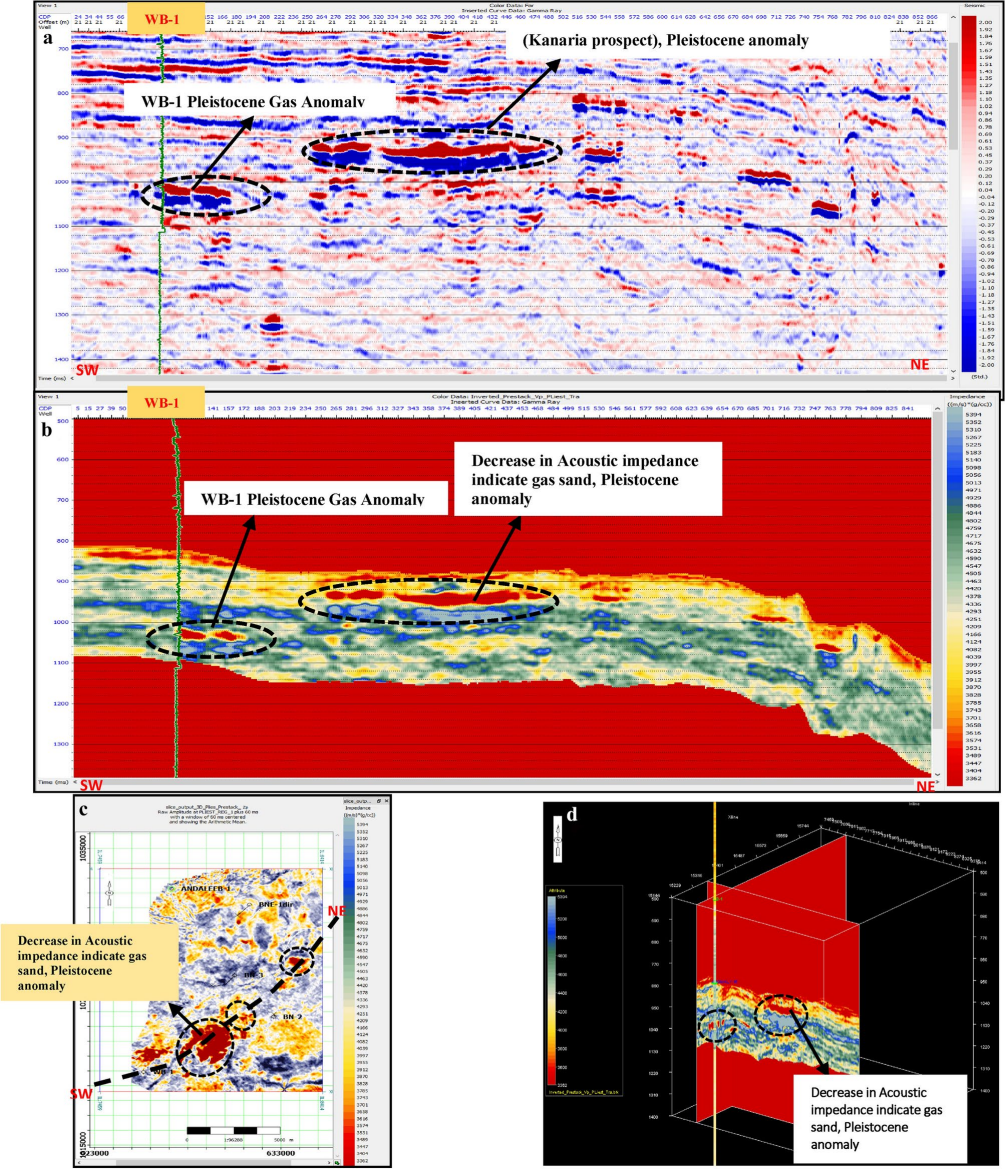
(**a**) Far SW-NE seismic section at Pleistocene anomaly (El Wastani Fm.). (**b**) SW-NE seismic section showing Zp Pre-Stack inversion model, Pleistocene anomaly (El Wastani Fm.). (**c**) Time Slice extracted for Zp Pre-Stack inversion model, extracted at Pleistocene gas sand anomaly (El Wastani Fm.). (**d**) 3D Volume showing Zp Pre-Stack inversion model, Pleistocene anomaly (El Wastani Fm.).

**Fig. 18:**
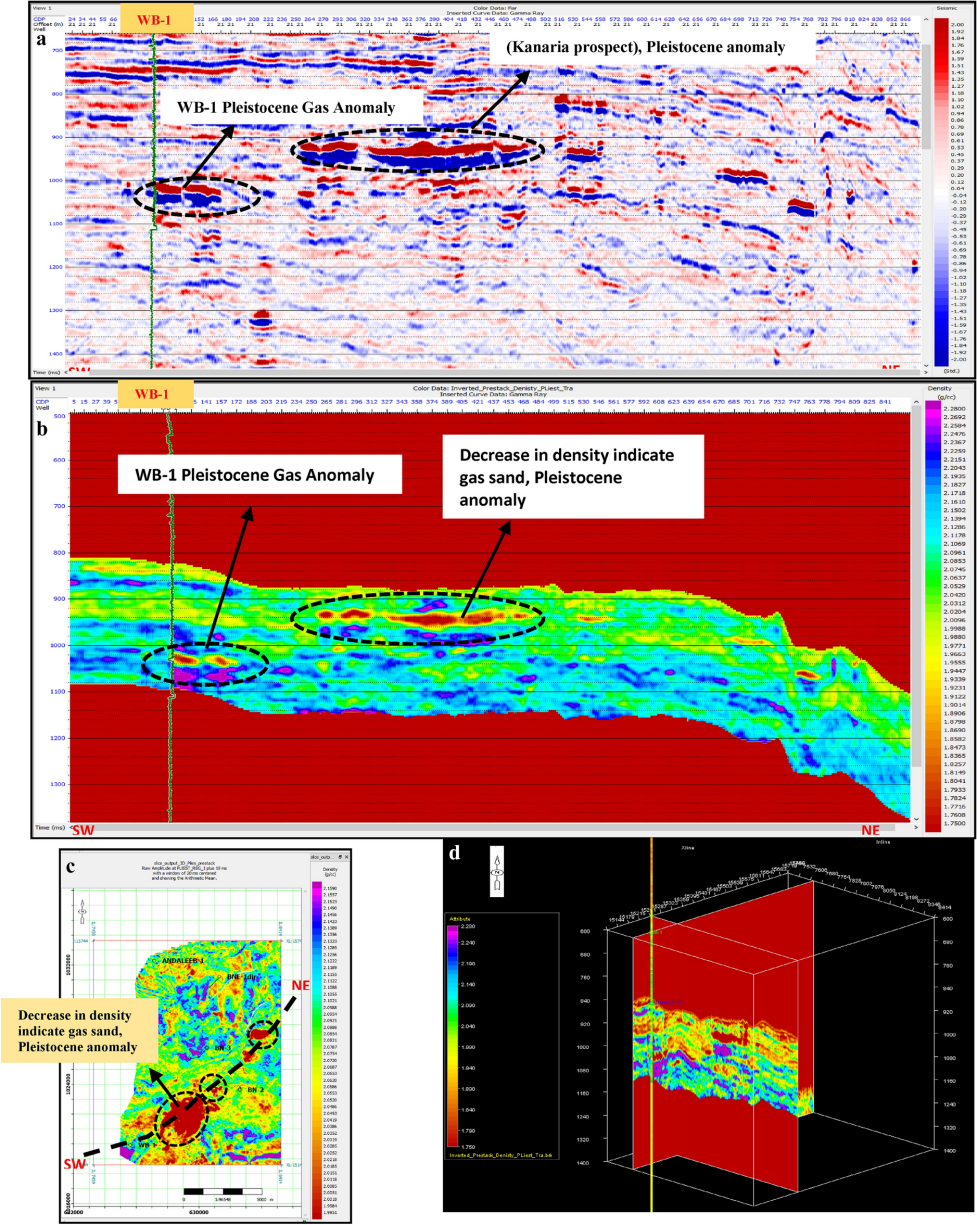
(**a**) Far SW-NE seismic section at Pleistocene anomaly (El Wastani Fm.). (**b**) SW-NE seismic section showing Density Pre-Stack inversion model, Pleistocene anomaly (El Wastani Fm.). (**c**) Time Slice extracted for Density Pre-Stack inversion model, extracted at Pleistocene gas sand anomaly (El Wastani Fm.). (**d**) 3D Volume showing Density Pre-Stack inversion model, Pleistocene anomaly (El Wastani Fm.).

**Fig. 19:**
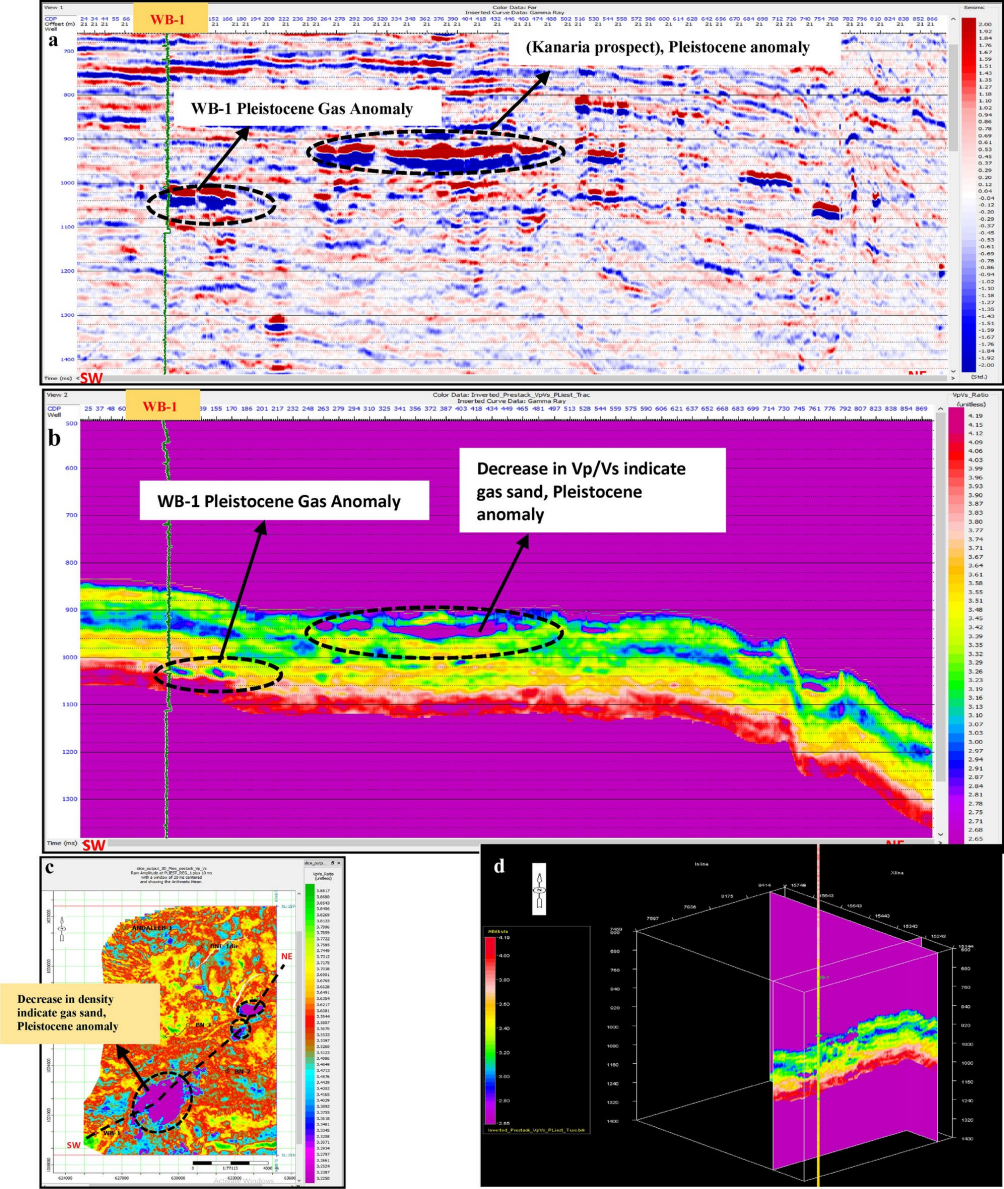
(**a**) Far SW-NE seismic section at Pleistocene anomaly (El Wastani Fm.). (**b**) SW-NE seismic section showing Vp/Vs ratio Pre-Stack inversion model, Pleistocene anomaly (El Wastani Fm.). (**c**) Time Slice extracted for Vp/Vs ratio Pre-Stack inversion model, extracted at Pleistocene gas sand anomaly (El Wastani Fm.). (**d**) 3D Volume showing Vp/Vs ratio Pre-Stack inversion model, Pleistocene anomaly (El Wastani Fm.).

**Fig. 20:**
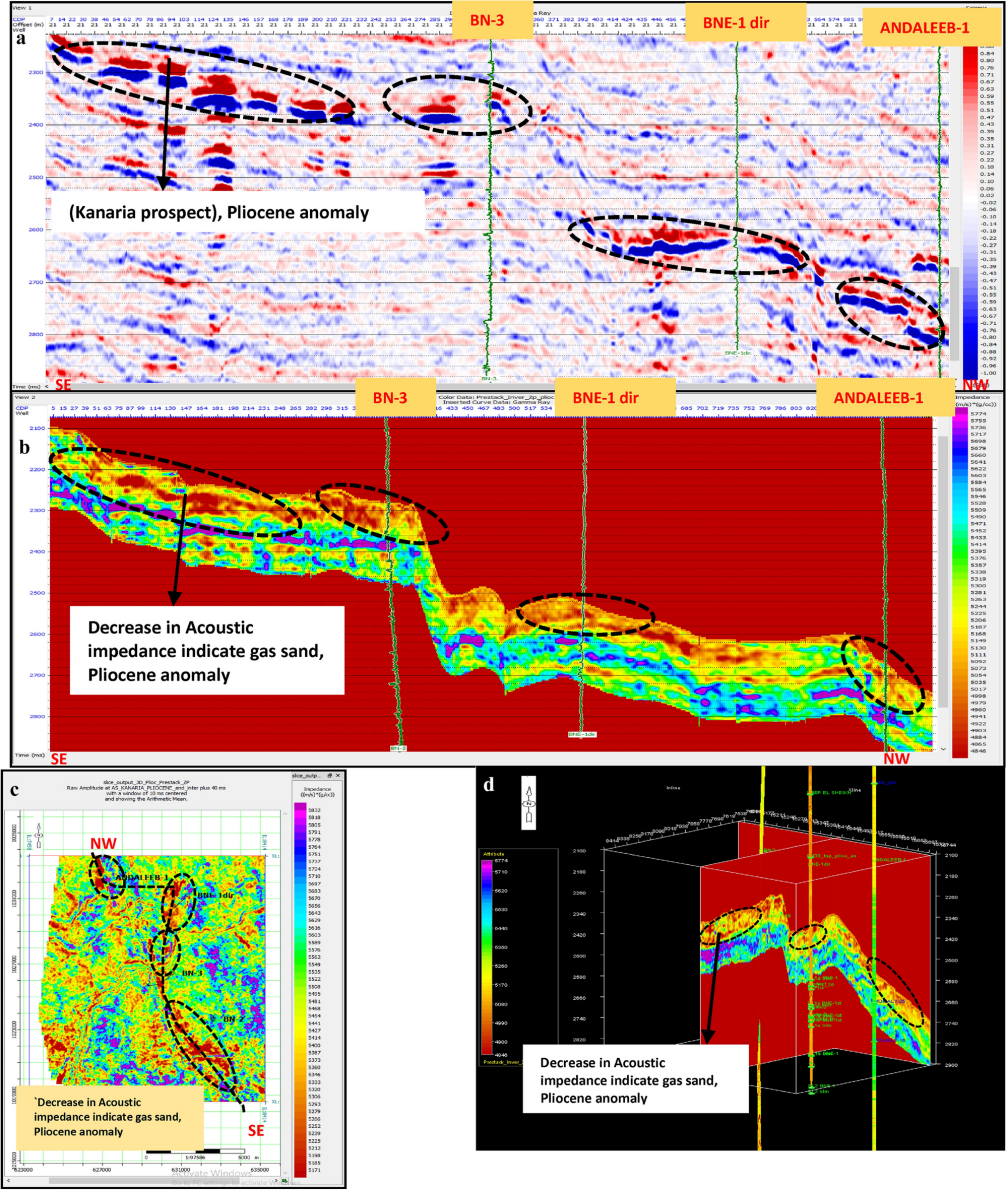
(**a**) Far SW-NE seismic section at Pliestocene anomaly (Kafr El Sheikh Fm.). (**b**) SW-NE seismic section showing Zp Pre-Stack inversion model, Kafr El-Sheikh Fm. (Pliocene Anomaly). (**c**) Time Slice extracted for Zp Pre-Stack inversion model, extracted at Kafr El-Sheikh Fm. (Pliestocene Anomaly). (**d**) 3D Volume showing Zp Pre-Stack inversion model, Pliocene anomaly (Kafr El-Sheikh Fm.).

**Fig. 21:**
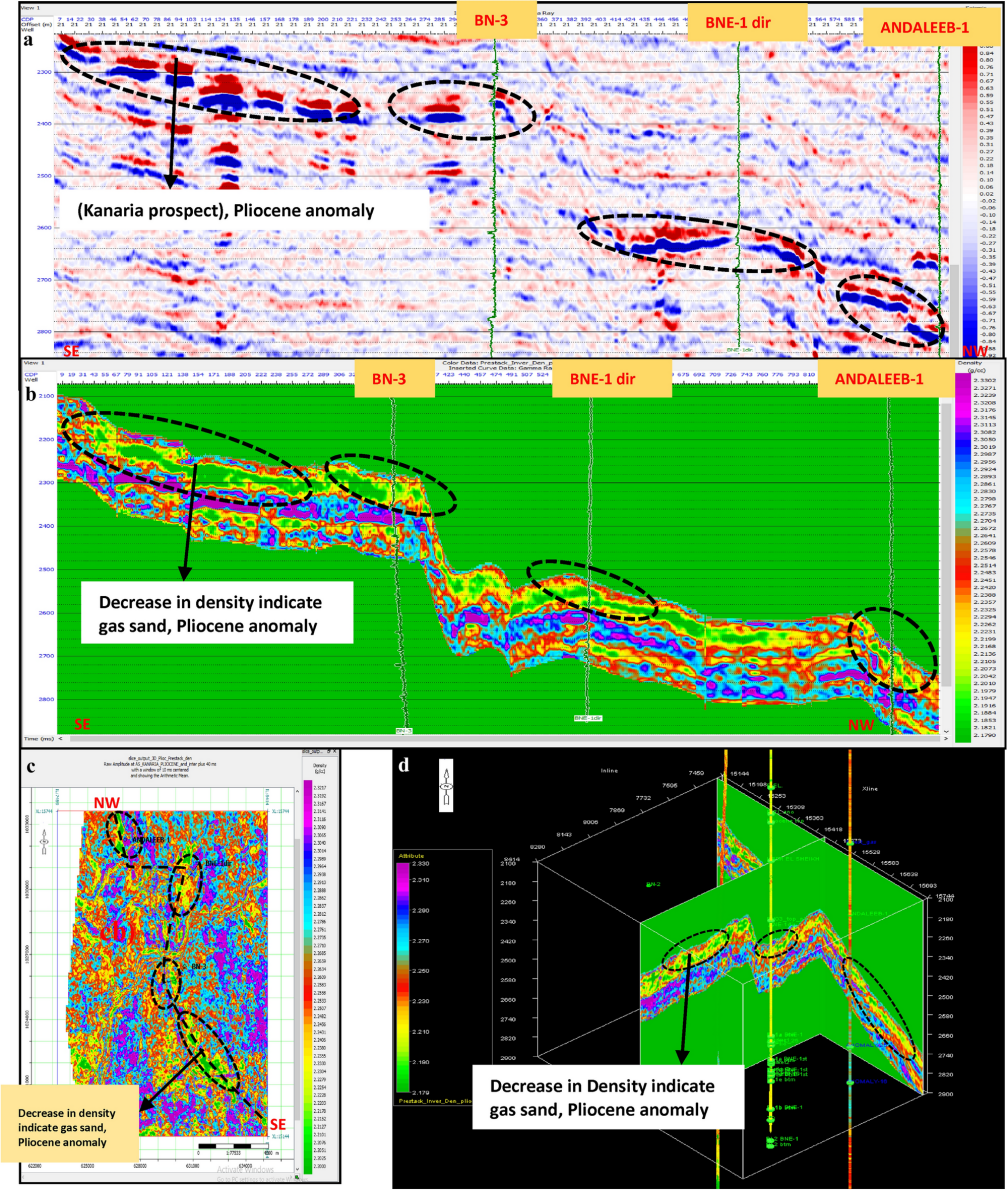
(**a**) Far SW-NE seismic section at Pliocene anomaly (Kafr El Sheikh). (**b**) SW-NE seismic section showing Density Pre-Stack inversion model, Kafr El-Sheikh Fm. (Pliocene Anomaly). (**c**) Time Slice extracted for Density Pre-Stack inversion model, extracted at Kafr El-Sheikh Fm. (Pliocene Anomaly). (**d**) 3D Volume showing Density Pre-Stack inversion model, Kafr El-Sheikh Fm. (Pliocene Anomaly).

**Fig. 22:**
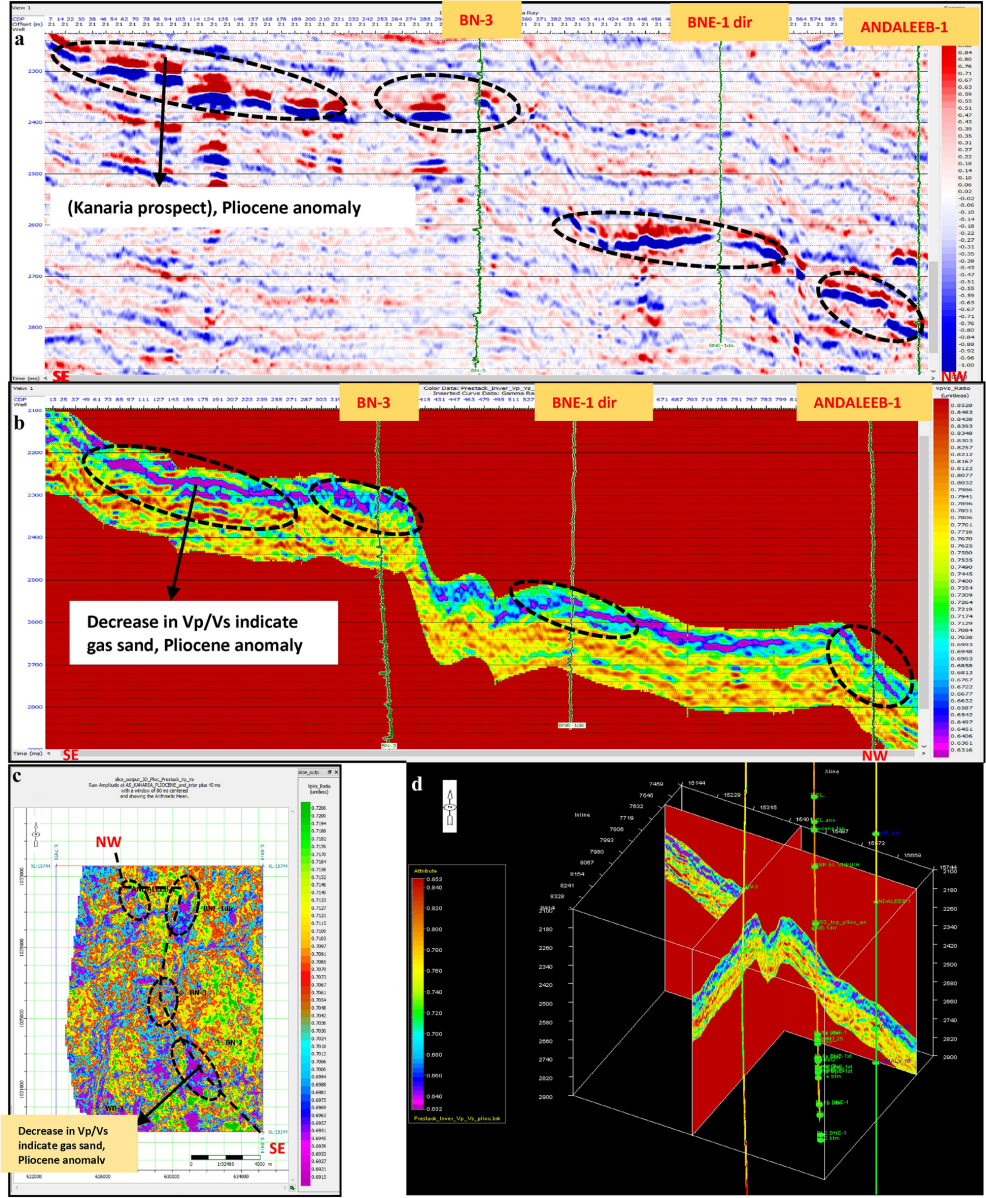
(**a**) Far SW-NE seismic section at Pliocene anomaly (Kafr El Sheikh). (**b**) SW-NE seismic section showing Vp/Vs ratio Pre-Stack inversion model, Kafr El-Sheikh Fm. (Pliocene Anomaly). (**c**) Time Slice extracted for Vp/Vs ratio Pre-Stack inversion model, extracted at Kafr El-Sheikh Fm. (Pliocene Anomaly). (**d**) 3D Volume showing Vp/Vs ratio Pre-Stack inversion model, Kafr El-Sheikh Fm. (Pliocene Anomaly).

Pre-stack inversion was applied to both anomalies across three volumes: Primary Acoustic Impedance (Zp), density, and Vp/Vs ratio. The first anomaly, corresponding to the Pleistocene (El Wastani Fm.), highlights the top and base gas sand outlined in black (Figs. 17, 18, 19). The second anomaly pertains to the Middle Pliocene (Kafr El Sheikh Fm.) (Figs. 20, 21, 22).

### Extended elastic impedance (EEI), alternative methods, and validation

#### Extended elastic impedance (EEI) analysis

Using an approximate linear formulation of the Zoeppritz equations, the EEI inversion method calculates acoustic impedance in the pre-stack domain at varying incidence angles. According to Connolly^60^, this method provides a reliable foundation for calibrating and inverting angle stacks. The elastic inversion transforms the pre-stack data into extended impedance, then computes an angle-dependent weighted combination of V_p_, V_s_ and density as an EEI seismic characteristic^25^. The optimization of petrophysical factors, such as shale volume, water saturation, and porosity, can lead to the identification of optimal ranges through cross-correlation of the EEI curves^16,61–66^.

The gradient stack sections and intercept sections from the AVO (Amplitude Versus Offset) assessment are combined to compute the inversion stage EEI reflections at specific angles for any parameter value^25^. The Shuey two-term approximation applied to pre-stack gathers allows for the determination of the AVO’s intercept and gradient [Shuey, 1985]. Accuracy is crucial as the angular range of the offset curve is differently defined by each of the intercept and gradient features. To invert the EEI reflectivity volume and yield a target component, a post-stack model-based inversion technique should be employed.

A model-driven approach integrates synthetic data with real seismic data using a simplified initial input model. Synthetic data are generated by convolutioning the reflectivity with a wavelet, and then compared with F

#### Results of EEI inversion

The EEI analysis aimed to determine the optimal operational angles based on reservoir target variables. EEI logs were created using target logs from Well WB-1 (Pleistocene anomaly, El-Wastani Fm.) and Well ANDALEEB-1 (Pliocene anomaly, Kafr El-Sheikh Fm.) to investigate cross-correlation. The EEI logs were generated within a range of − 90° to + 90°. The final results include:

Pleistocene Prospect (El Wastani Fm.) at WB-1: Optimal angles identified are χ = 75°, χ = 77°, and χ = 76°.

Middle Pliocene Prospect (Kafr El Sheikh Fm.) at ANDALEEB-1: Optimal angles identified are χ = − 32°, χ = − 36°, and χ = − 38°.

**Fig. 23:**
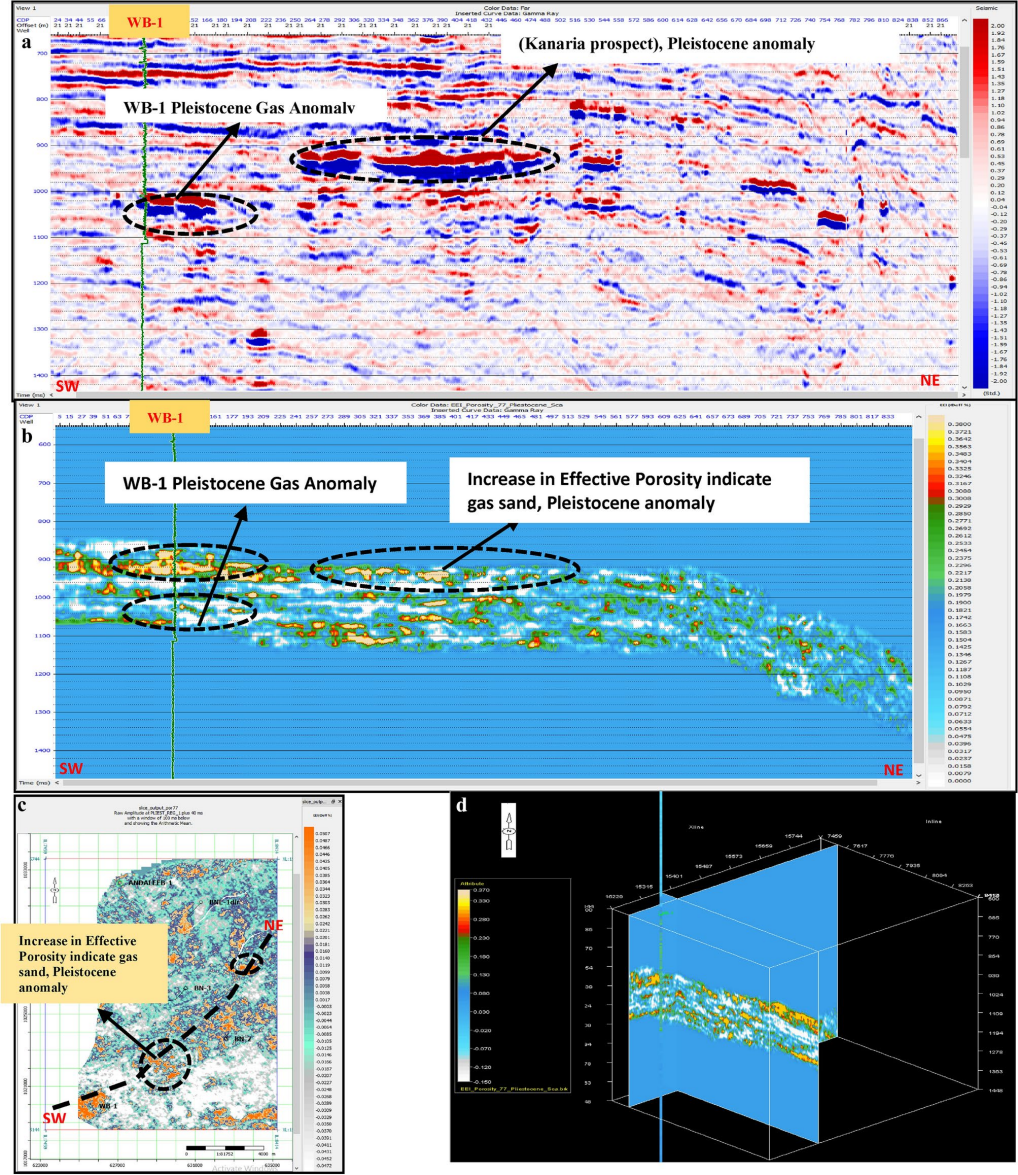
(**a**) Far SW-NE seismic section at Pleistocene anomaly (El Wastani Fm.). (**b**) SW-NE EEI inverted effective Porosity (Φeff), Pleistocene anomaly. (**c**) Time Slice extracted for EEI inverted effective Porosity (Φeff) at El Wastani Fm. (Pleistocene anomaly). (**d**) 3D Volume showing EEI inverted effective Porosity (Φeff), Pleistocene anomaly (El Wastani Fm.).

**Fig. 24:**
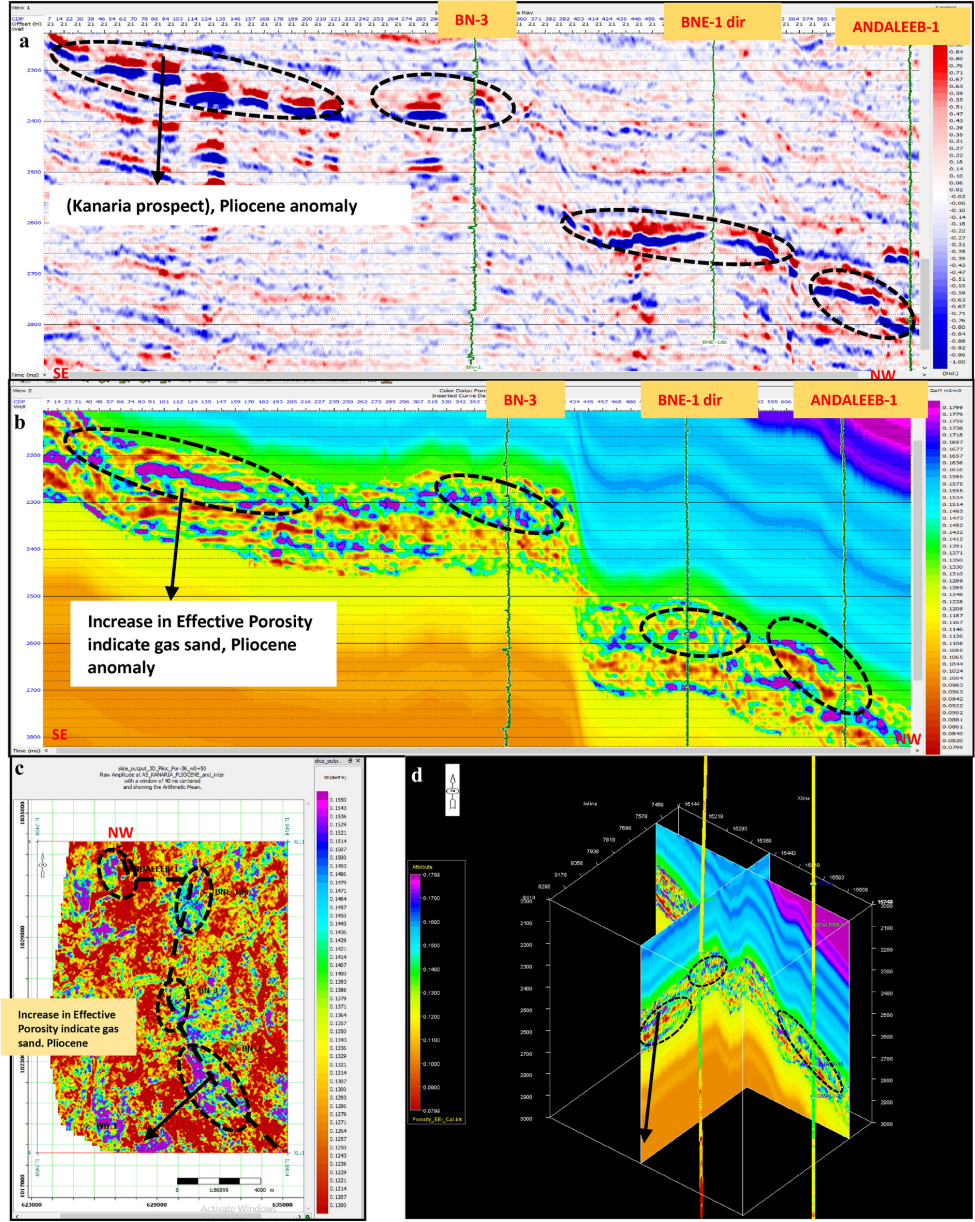
(**a**) Far SW-NE seismic section at Pliocene anomaly (El Wastani Fm.). (**b**) SW-NE seismic section showing EEI inverted effective Porosity, Kafr El-Sheikh Fm. (Pliocene Anomaly). (**c**) Time Slice extracted from EEI inverted effective Porosity (Φeff) at Kafr El-Sheikh Fm. (Pliocene Anomaly). (**d**) 3D Volume showing EEI inverted effective Porosity (Φeff) at Pliocene anomaly (Kafr El-Sheikh Fm.).

These optimal angles were used to reveal the following volumes:

Porosity Volume (Figs. 23 and 24)

**Fig. 25:**
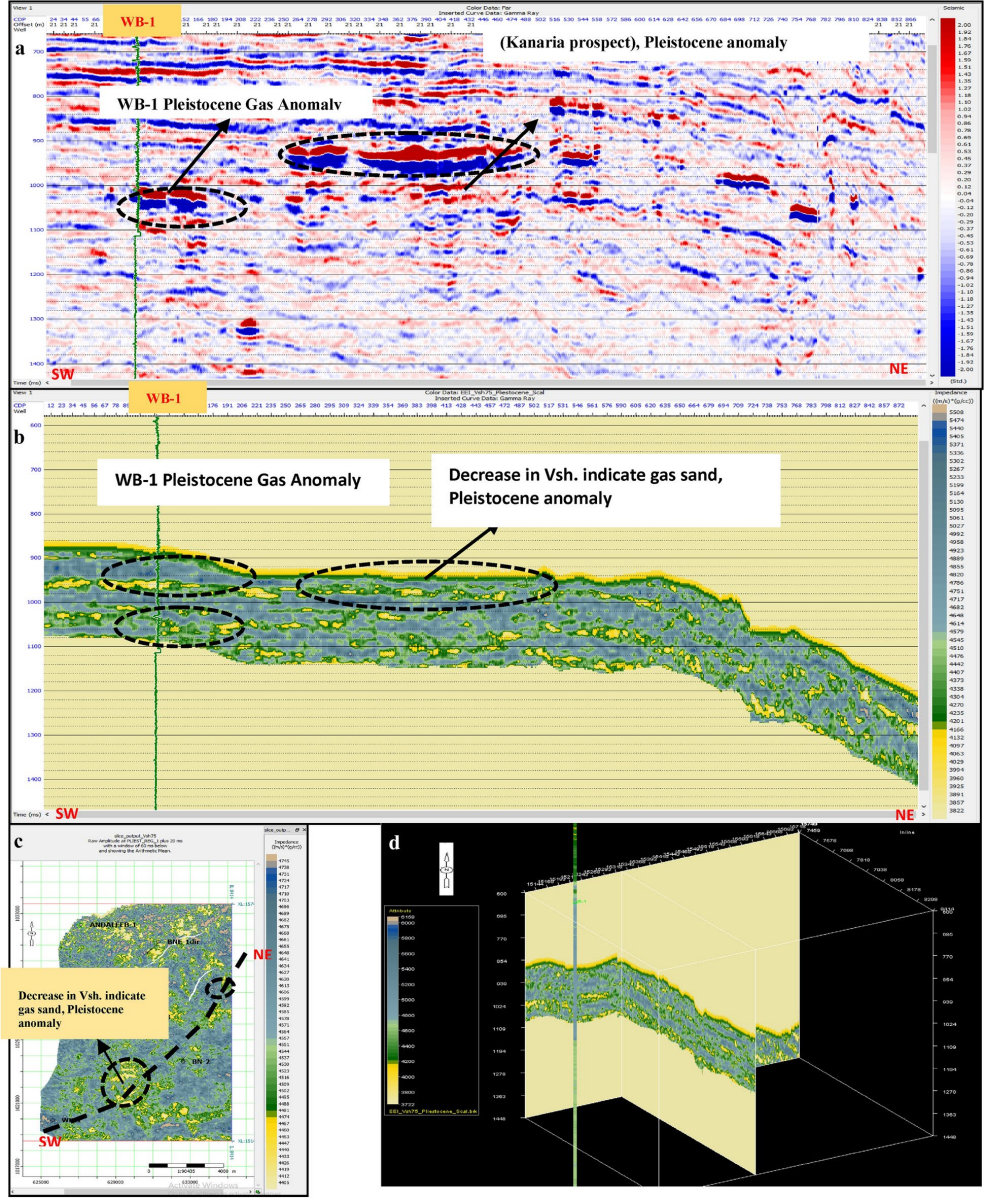
(**a**) Far SW-NE seismic section at Pleistocene anomaly (El Wastani Fm.). (**b**) SW-NE EEI inverted Shale volume (Vsh), Pleistocene anomaly (El Wastani Fm.). (**c**) Time Slice extracted from EEI inverted Shale volume at El Wastani Fm. (Pleistocene anomaly). (**d**) 3D Volume showing EEI inverted Shale volume (Vsh), Pleistocene anomaly (El Wastani Fm.).

**Fig. 26:**
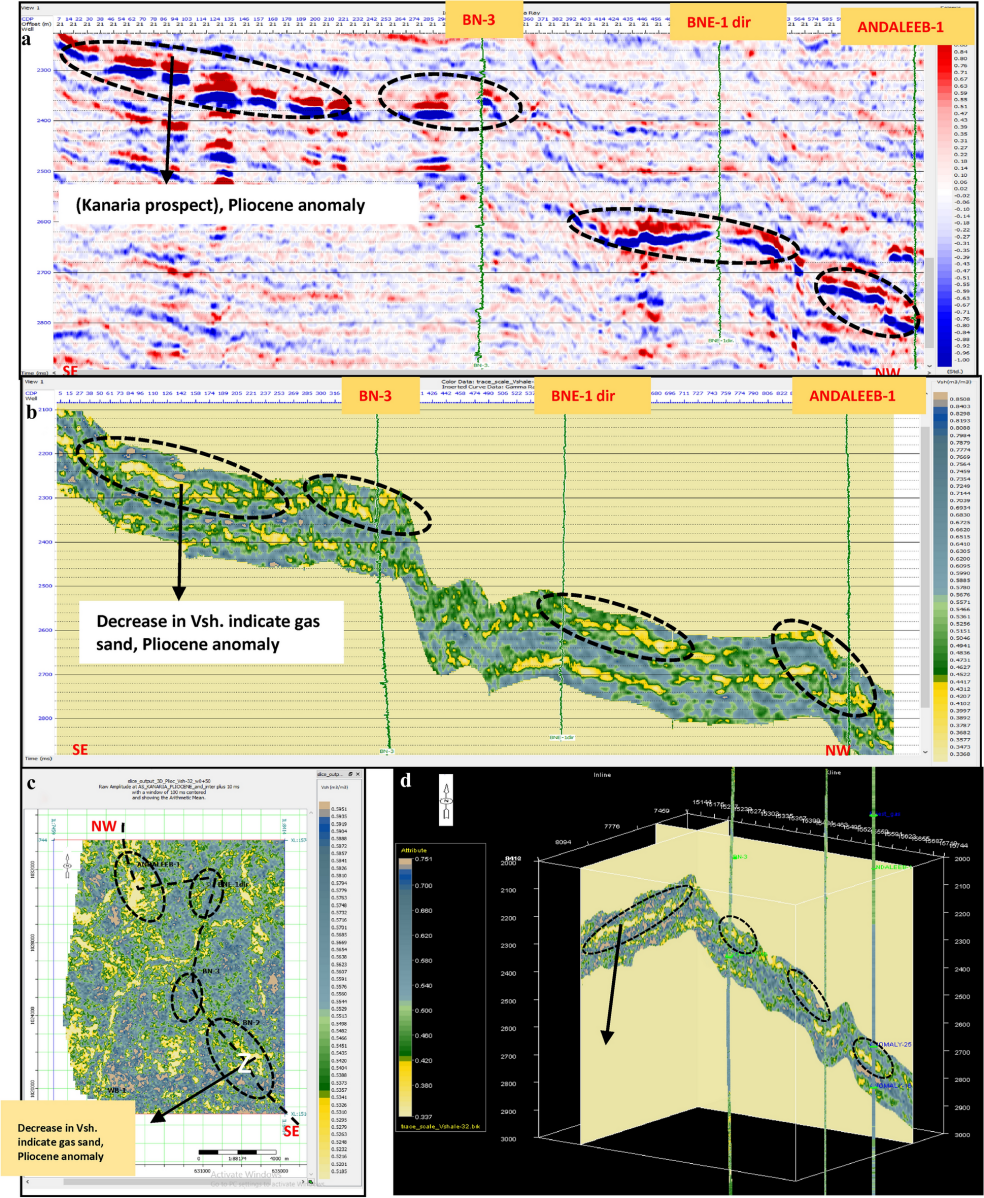
(**a**) Far SW-NE seismic section at Pliocene anomaly (Kafr El Sheikh Fm.). (**b**) SW-NE seismic section showing EEI inverted Shale volume (Vsh), Kafr El-Sheikh Fm. (Pliocene Anomaly). (**c**) Time Slice extracted from EEI inverted Shale volume at Kafr El-Sheikh Fm. (Pliocene Anomaly). (**d**) 3D Volume showing EEI inverted Shale volume (Vsh) at Pliocene anomaly (Kafr El-Sheikh Fm.).

Shale Content Volume (VSh) (Figs. 25 and 26)

**Fig. 27:**
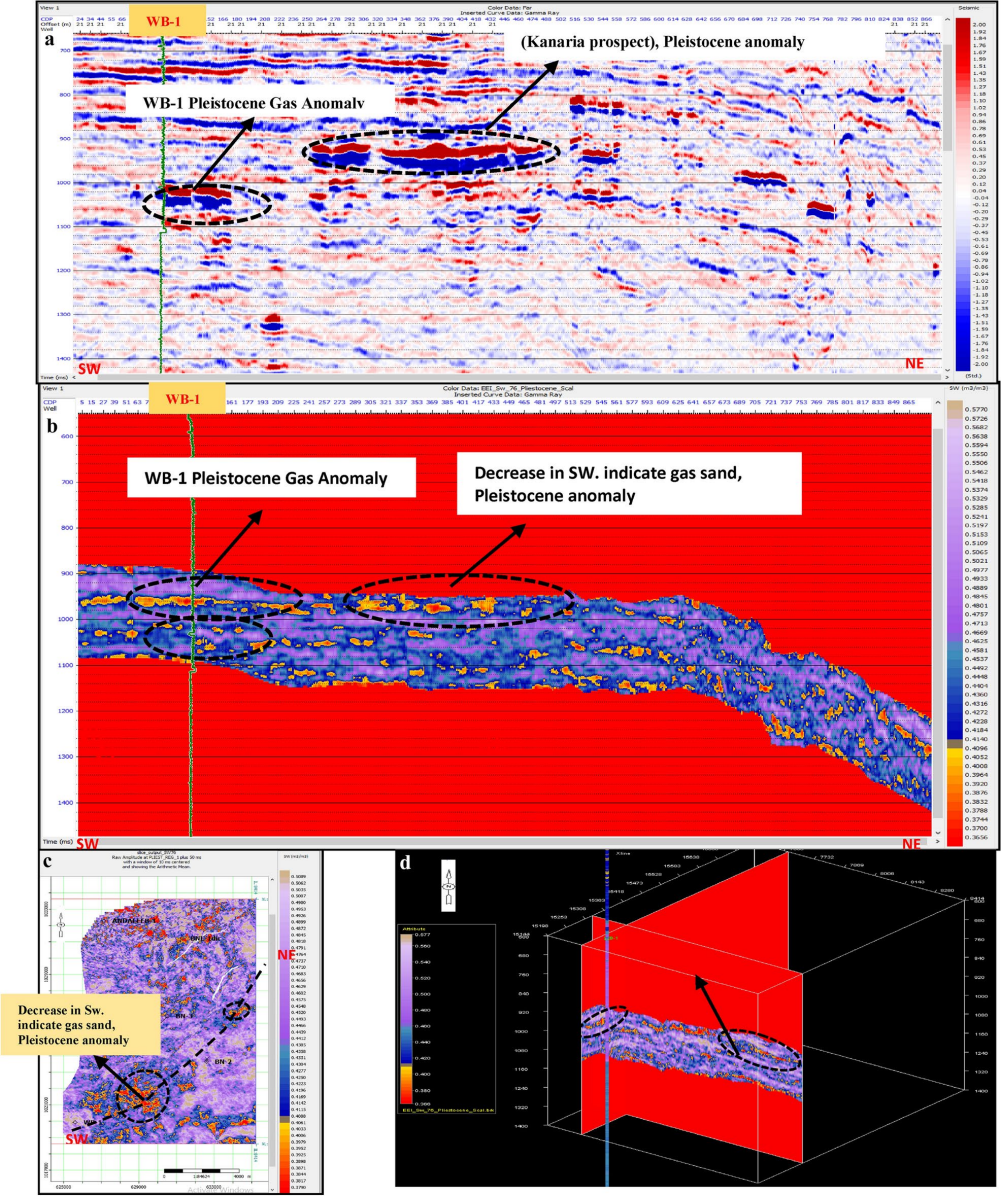
(**a**) Far SW-NE seismic section at Pleistocene anomaly (El Wastani Fm.). (**b**) SW-NE EEI inverted Water saturation (Sw), Pleistocene anomaly. (**c**) Time Slice extracted from EEI inverted Water saturation (Sw) at El Wastani Fm. (Pleistocene anomaly). (**d**) 3D Volume showing EEI inverted Water saturation (Sw), Pleistocene anomaly (El Wastani Fm.).

**Fig. 28:**
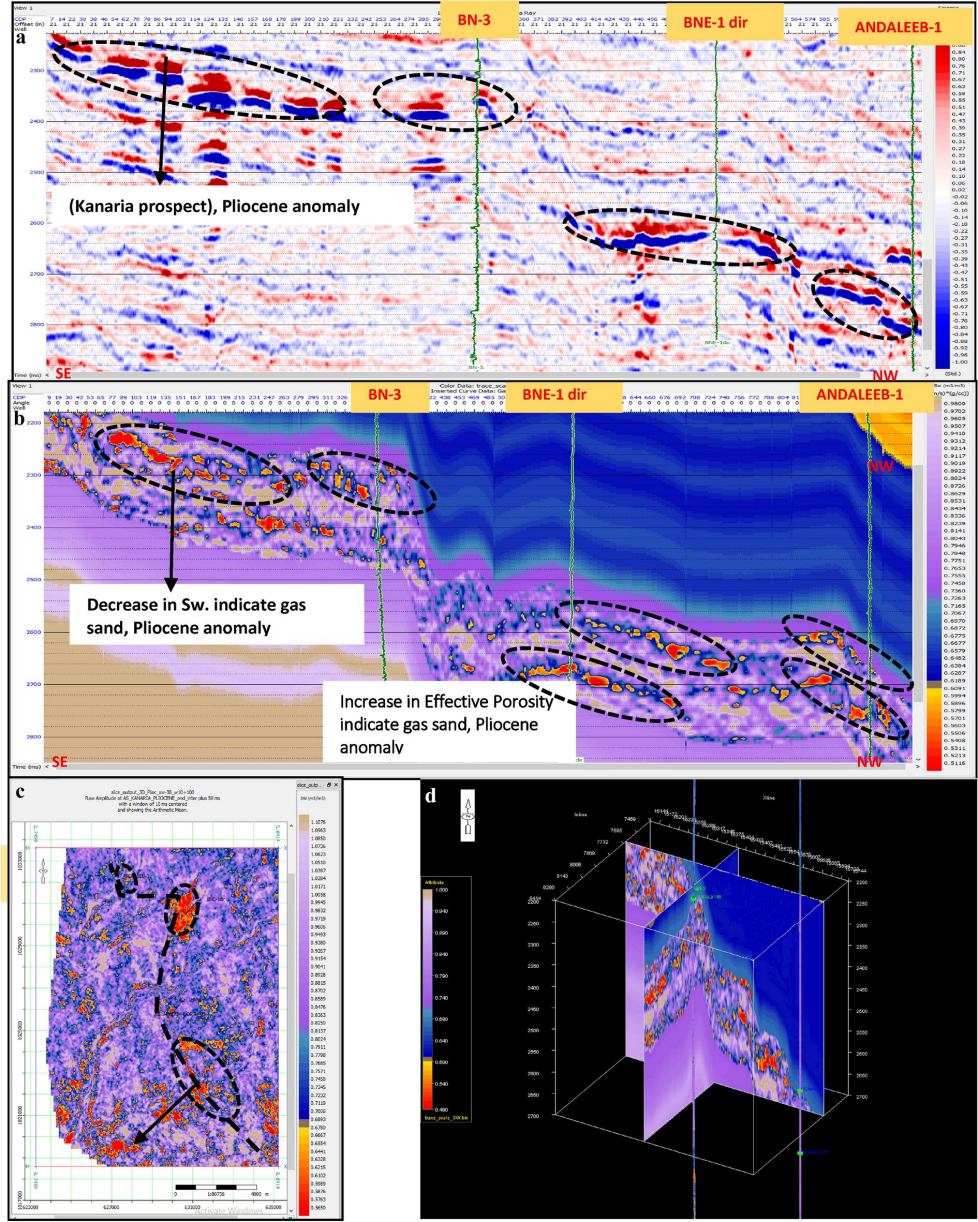
(**a**) Far SW-NE seismic section at Pliocene anomaly (Kafr El Shiekh Fm.). (**b**) SW-NE seismic section showing EEI inverted Water saturation (Sw), Kafr El-Sheikh Fm. (Pliocene Anomaly). (**c**) Time Slice extracted from EEI inverted effective Porosity (Φeff) at Kafr El-Sheikh Fm. (Pliocene Anomaly). (**d**) 3D Volume showing EEI inverted Water saturation (Sw) at Pliocene anomaly (Kafr El-Sheikh Fm.).

Water Saturation Volume (Figs. 27 and 28).

#### Alternative methods and validation

Alternative Techniques: In addition to pre-stack inversion and EEI, methods such as post-stack inversion, sparse spike inversion, and multi-attribute analysis offer complementary insights. Post-stack inversion may reveal different subsurface aspects, while sparse spike inversion enhances resolution. Multi-attribute analysis integrates various seismic attributes for a comprehensive understanding^60,67^.

Validation Approaches: To validate findings, cross-referencing with additional well log data from nearby wells helps confirm seismic interpretations and address discrepancies. Sensitivity analyses on key parameters and inputs assess how variations affect results, ensuring robustness^68^.

Practical Implications: Incorporating alternative methods and validation strategies enhances the reliability of results and provides a comprehensive guide for professionals in seismic data interpretation and hydrocarbon exploration.

### Risk assessment

Geological risk assessment is used to evaluate whether a proposed drilling target is likely to be successful or unproductive based on available geological parameters^60,67,68^. The primary source of categorization uncertainty stems from the geological risk that the project may turn out dry. This method addresses the ambiguity in a multidimensional space and depends on the availability of relevant data.

The evaluation of the probability of success (POS) considers risk factors such as Play, Local Reservoir Variations, Trap, Local Seal, and Charge. Inversion analysis is a crucial tool in confirming the presence of hydrocarbons for both prospective anomalies, which enhances the overall probability of success. The main risks recently identified include:

Timing: The period necessary for kerogen maturation may be insufficient for converting kerogen into hydrocarbons.

Temperature: The amount of heat required for the conversion of kerogen into hydrocarbons might be inadequate.

**Fig. 29:**
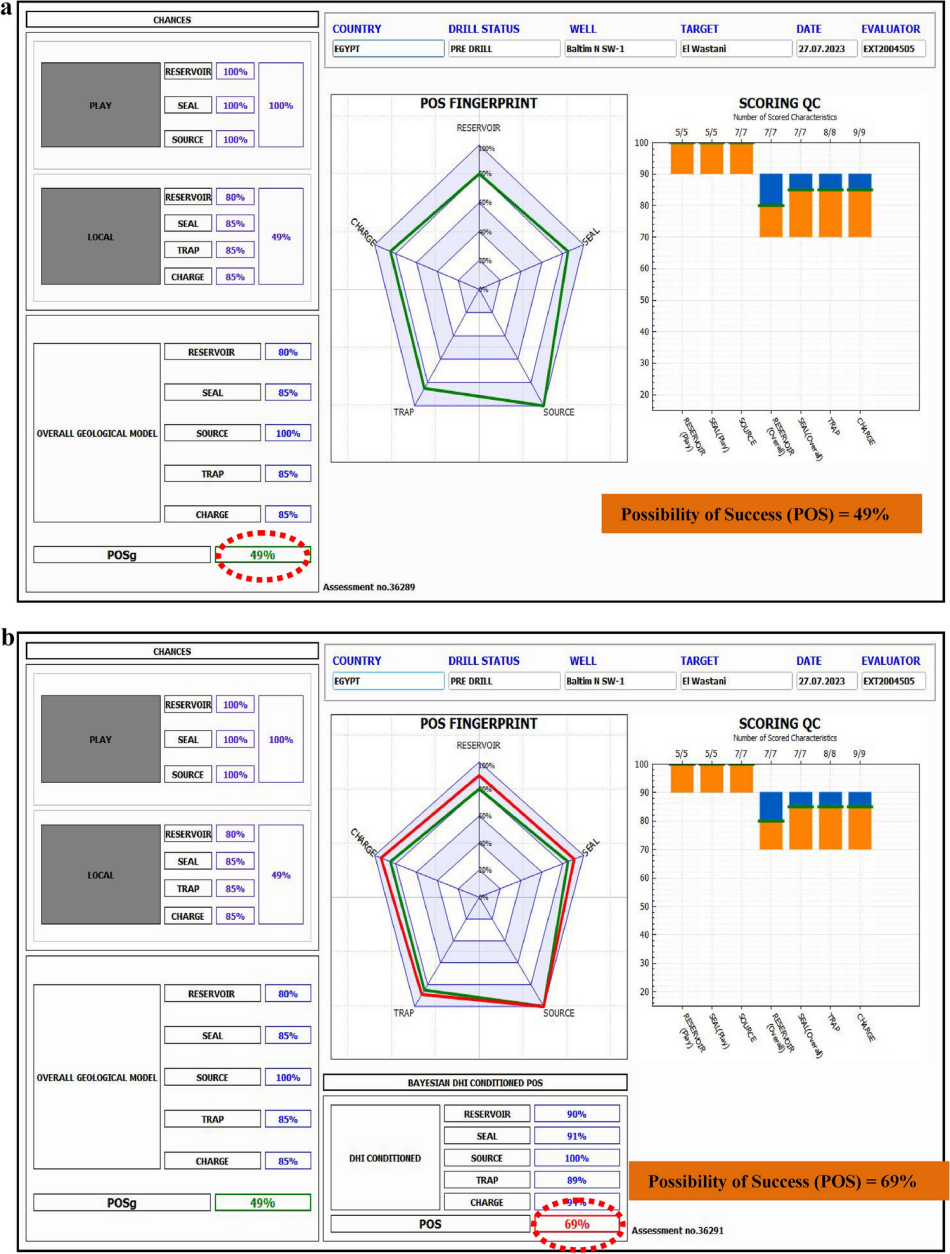
(**a**) Possibility of Success, before applying Inversion technique, Pleistocene prospect anomaly (El-Wastani Fm.). (**b**) Possibility of Success, after applying Inversion technique, Pleistocene prospect anomaly (El-Wastani Fm.).

These risks are particularly relevant concerning the depth of the Pliocene target. The POS estimates are:

For the El Wastani Fm. (Pleistocene Anomaly): 49% to 69% (Fig. 29).

**Fig. 30:**
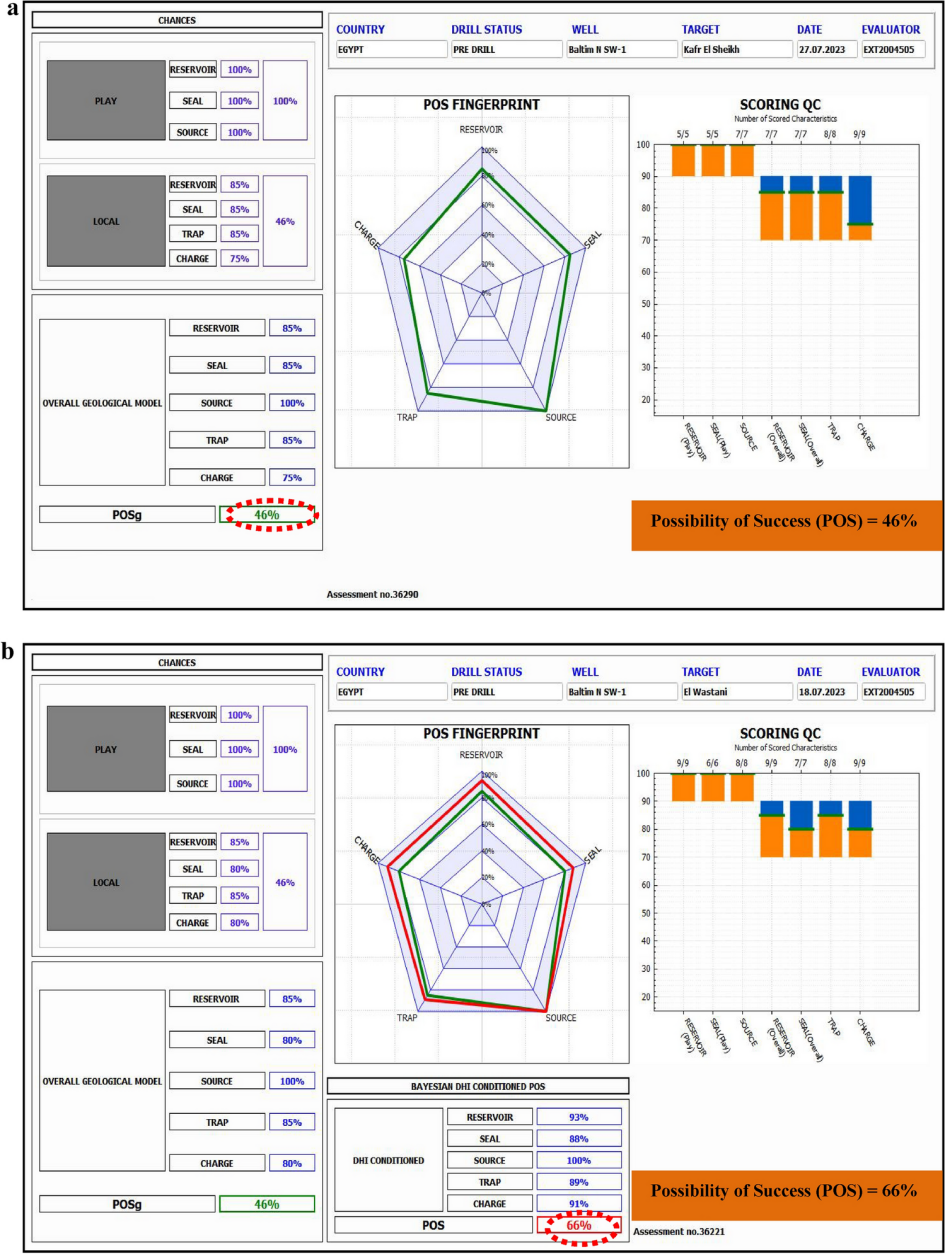
(**a**) Possibility of Success, before applying Inversion technique, Pliocene prospect anomaly (Kafr El-sheikh Fm.). (**b**) Possibility of Success, after applying Inversion technique, Pliocene prospect anomaly (Kafr El-sheikh Fm.).

For the Kafr El Sheikh Fm. (Middle Pliocene Anomaly): 46 to 66% (Fig. 30).

Inversion analysis significantly contributes to assessing hydrocarbon presence and mitigating associated risks.

## Conclusion and recommendations

This research significantly advances the identification of rock properties in reservoir gas sands using a combination of post- and pre-stack inversion with Extended Elastic Impedance (EEI) analysis. The study focused on two prospective anomalies, the Pleistocene and Pliocene anomalies—revealing key attributes such as porosity, shale volume, and water saturation. Inversion analysis has proven to be a crucial tool for confirming hydrocarbon presence and evaluating petroleum system elements (source, reservoir, and seal rocks), thereby enhancing the Probability of Success (POS).

The application of cross-plots, such as AI versus SI, has demonstrated that low AI and SI values are indicative of hydrocarbon presence in both anomalies. Additionally, the relationship between AI and the Vp/Vs ratio, where low acoustic impedance (AI) combined with high Vp/Vs suggests a higher likelihood of gas, underscores the importance of well log quality control for identifying gas anomalies. The study’s approach integrates traditional methods, such as Far map amplitude analysis, with advanced inversion techniques, including EEI, to provide a quantitative reservoir assessment.

The findings confirm a strong correlation between essential reservoir characteristics, porosity, shaliness, and water saturation—and EEI at various χ angles. By establishing optimal φ angles for each reservoir feature and constructing EEI volumes at these ideal angles, the study provides valuable evidence for reservoir characterization. EEI volumes can be utilized as secondary inputs in volume calculations and risk assessments, increasing the likelihood of successful hydrocarbon discovery. This approach also aids in identifying promising drilling locations for future exploration.

Based on the findings from the Pleistocene and Pliocene anomalies in the Nile Delta, several recommendations and broader implications for future research and exploration can be made:

Application of Methodologies: The seismic inversion techniques and petrophysical analyses demonstrated in this study, including pre-stack inversion and Extended Elastic Impedance (EEI), are recommended for application in other sedimentary basins with similar geological settings. These methods provide valuable insights into the identification and characterization of hydrocarbon resources, which can be beneficial in diverse geological contexts.

Adaptation to Other Regions: The approach used to distinguish between different stratigraphic anomalies and assess their impact on hydrocarbon accumulation can be adapted to other regions with comparable geological and geophysical characteristics. This adaptation can facilitate a better understanding of subsurface conditions and improve exploration strategies in various contexts.

Broader Implications: The insights gained regarding the impact of gas effects on seismic responses and the application of detailed petrophysical analysis can inform exploration and interpretation efforts in different regions. Future studies should consider these methodologies to enhance the exploration and characterization of hydrocarbon resources beyond the Nile Delta.

Specific Recommendations for Baltim Area: Drilling for the Kanaria prospect (Baltim N SW-1) is recommended to explore additional potential within the Baltim North exploration lease, particularly within the El Wastani and Kafr El Sheikh Formations. The EEI results for these anomalies reveal high amplitude with a far offset, high effective porosity, smaller shale volume, and lower water saturation. This information should guide the selection of optimal drilling locations for Baltim N SW-1, aiming to uncover further prospects in the region.

By incorporating these recommendations and applying the methods to other geological settings, the relevance and impact of this study can be extended to a wider audience, benefiting the broader field of seismic data interpretation and hydrocarbon exploration.

## Discussion

Geological Setting and Vp/Vs Ratios: The net pay and Vp/Vs ratios in the Pleistocene and Pliocene formations are influenced by their respective geological settings. The Pleistocene formation, characterized by a braided channel system and found in localized shallow sections, often exhibits higher velocities due to the higher density and compactness of sediments in such channels. In contrast, the Pliocene formation, located in intermediate sections, may show lower velocities due to different sedimentary processes, such as finer-grained deposits and potentially higher porosity, which affect the seismic response. This geological variation leads to differing Vp/Vs ratios even in gas-bearing sands. The channel’s influence on velocity and net pay reflects the complex interplay between sedimentary facies and seismic attributes.

This study focuses on integrating post- and pre-stack inversion techniques with Extended Elastic Impedance (EEI) analysis to characterize gas-bearing sand reservoirs in the Pleistocene and Pliocene anomalies of the Nile Delta. The results are compared with those from the study by Ali et al.^22^, which explored EEI for delineating gas-bearing sands in the Saffron Gas Field, Offshore Nile Delta, Egypt.

### Comparison with Ali et al.^22^

#### Seismic inversion techniques

Pre-Stack Inversion: Both studies validate the efficacy of pre-stack inversion in enhancing the resolution of gas reservoirs. Our findings confirm that, similar to Ali et al.^22^, pre-stack inversion improves the accuracy of reservoir characterization by leveraging data across a range of angles. This method proves essential for identifying hydrocarbon-bearing zones with greater precision.

#### Extended elastic impedance (EEI)

Methodology and Outcomes: The EEI approach in our study aligns with the methodology described by Ali et al.^22^. We observed optimal angles for EEI analysis, with χ = 75°, 77°, and 76° for the Pleistocene anomaly, and χ = -32°, -36°, and -38° for the Pliocene anomaly. These findings corroborate Ali et al.’s results, which demonstrated the effectiveness of EEI in distinguishing between gas-bearing and non-gas-bearing sands. The ability of EEI to convert intercept and gradient volumes into fluid and lithology 3D volumes, as achieved by Ali et al., is also evident in our study, reinforcing EEI’s role in reservoir characterization.

#### Quality control and cross-plot analysis

Well Log Quality: Our study highlights the importance of well log quality control, a point also emphasized by Ali et al.^22^. Cross-plots such as AI versus SI and AI versus Vp/Vs ratio were crucial in identifying gas anomalies. The consistency between our results and those of Ali et al. underscores the reliability of these cross-plot methods in distinguishing hydrocarbon-bearing zones and validating the quality of well logs.

#### Reservoir characterization

Application and Results: Similar to Ali et al., our study demonstrates that EEI effectively characterizes gas reservoirs. By comparing gas wells and using various inversion techniques, including traditional methods and EEI, we confirmed the strong association between reservoir characteristics (porosity, shaliness, water saturation) and EEI values at different χ angles. The methodology from Ali et al., which involved deriving petrophysical properties from EEI reflectivity volumes and validating them with blind wells, aligns with our approach and results.

#### Challenges and future directions

Both our study and Ali et al. face challenges such as seismic resolution limitations and lateral variations affecting data accuracy. These issues impact the precision of inversion results and highlight the need for continued advancements in inversion techniques and data acquisition methods. Future research should focus on improving the resolution of seismic data and addressing the limitations identified in both studies to enhance the accuracy of reservoir characterization.

#### Broader implications

Our findings, combined with the results from Ali et al.^22^, support the broader application of seismic inversion and EEI techniques in sedimentary basins. The successful application in the Nile Delta suggests that these methods can be adapted to other regions with similar geological and geophysical settings, potentially improving hydrocarbon exploration and reservoir characterization in various contexts.

By integrating insights from Ali et al.^22^ and our own research, this discussion highlights the effectiveness and potential of EEI and seismic inversion techniques, providing a comprehensive understanding of their application in gas reservoir delineation.

## Data Availability

The data that support the findings of this study are available on request from the corresponding first author [Ali EL-SAYED]. The data are not publicly available due to [restrictions, because of their containing information that could compromise the privacy of research participants.

## Well identifiers

BN-3: Baltim North-3 well
BNE-1dir: Baltim North East-1 directional
Wb-1: West Baltim-1

## Organizations

FGPC: Egyptian General Petroleum Corporation
IEOC: International Egyptian Oil Company

## Geological and petrophysical properties

V_sh_: Shale volume
Φ_T_: Total porosity
Φ_E_: Effective porosity
Φ: Porosity (as fraction)
Sw: Water saturation
Vp: P-wave velocity
Vs: S-wave velocity
